# LncRNA H19 initiates microglial pyroptosis and neuronal death in retinal ischemia/reperfusion injury

**DOI:** 10.1038/s41418-019-0351-4

**Published:** 2019-05-24

**Authors:** Peixing Wan, Wenru Su, Yingying Zhang, Zhidong Li, Caibin Deng, Jinmiao Li, Nan Jiang, Siyu Huang, Erping Long, Yehong Zhuo

**Affiliations:** 1 State Key Laboratory of Ophthalmology, Zhongshan Ophthalmic Center, Sun Yat-sen University, 510060 Guangzhou, China; 2 Department of Molecular, Cellular, and Developmental Biology, University of Michigan, Ann arbor, MI 48109 USA

**Keywords:** Epigenetics, Cell death and immune response, Inflammasome, Microglia

## Abstract

Ischemia-reperfusion (I/R) is a common pathology when the blood supply to an organ was disrupted and then restored. During the reperfusion process, inflammation and tissue injury were triggered, which were mediated by immunocytes and cytokines. However, the mechanisms initiating I/R-induced inflammation and driving immunocytes activation remained largely unknown. In this study, we identified long non-coding RNA (lncRNA)-H19 as the key onset of I/R-induced inflammation. We found that I/R increased lncRNA-H19 expression to significantly promote NLRP3/6 inflammasome imbalance and resulted in microglial pyroptosis, cytokines overproduction, and neuronal death. These damages were effectively inhibited by lncRNA-H19 knockout. Specifically, lncRNA-H19 functioned via sponging miR-21 to facilitate PDCD4 expression and formed a competing endogenous RNA network (ceRNET) in ischemic cascade. LncRNA H19/miR-21/PDCD4 ceRNET can directly regulate I/R-induced sterile inflammation and neuronal lesion in vivo. We thus propose that lncRNA-H19 is a previously unknown danger signals in the molecular and immunological pathways of I/R injury, and pharmacological approaches to inhibit H19 seem likely to become treatment modalities for patients in the near future based on these mechanistic findings.

## Introduction

Ischemia and reperfusion (I/R) is a common pathological condition characterized by an initial restriction of blood supply and followed by the subsequent restoration of perfusion. I/R injury are critically involved in a wide spectrum of pathological conditions [1], including stroke, heart attack, organ transplantation, hypovolemic shock, and retinal vascular occlusion. Although reperfusion is needed, restoration of blood flow and reoxygenation is frequently associated with a profound inflammatory response and an exacerbation of tissue injury [2]. Recent evidence suggests that various immunocytes and cytokines are intimately involved in all stages of the ischemic cascade [3]; however, the onset of I/R-induced inflammation remained unclear.

Long non-coding RNAs (lncRNAs) have been reported to participate in activation of immunocytes [4] and transmission of dangerous signal [5], which might play important role in I/R injury. Recently, the overexpression, deficiency or mutation of lncRNA genes has been implicated crucial in different pathologies, including autoimmune disease [6], sepsis [7], and cancers [8]. Therefore, lncRNAs emerge as a breakthrough of deciphering molecular mechanisms underlying I/R-induced inflammation and thus serve as a potential therapeutic target. Noteworthy, competing endogenous RNA network (ceRNET) is a promising module to facilitate lncRNAs function in complex pathologic conditions. The ceRNET facilitates coordination between lncRNAs and coding genes in various molecular signals by forming large-scale regulatory circuitries across the transcriptome [9] in biological and pathological processes [10–13]. However, the specific role of lncRNAs in I/R-induced injury and its functional mechanism remain largely unknown.

Our previous study indicated that when suffer from ischemic insult, injured tissues release danger signals that activated NOD-like receptor protein 3 (NLRP3) inflammasomes [14]. The activated inflammasomes subsequently cleave gasdermin D (GSDMD) protein and lead to pyroptosis. Pyroptosis, inflammatory programmed cell death, is critical for defenses against pathogenic micro-organisms and danger signals. However, excessive pyroptosis leads to sudden cell swelling and lysis, which causes massive release of cellular contents and thereby triggers strong pro-inflammatory responses [15]. Cleaved caspase-1, an inflammatory caspase, also directly processes pro-interleukin-1β (IL-1β) and pro-IL-18 into their mature forms on platform of NLRP3 inflmmasome complex [16]. Importantly, NLRP3 inflammasome inhibition reduces the progression of sterile inflammatory diseases such as atherosclerosis [17], metabolic disease [18], autoinflammatory [19], and neuroinflammatory disorders [20]. In contrast, NLRP6 inflammsome was reported to defense against innate immunity and sterile inflammation via attenuating ROS-induced cytokines production and other mechanisms [21]. Therefore, key initiator of imbalanced activation between inflammsomes remains an urgent need to gain additional mechanistic insight that are triggered by ischemia and reperfusion and that could be exploited therapeutically.

The retina is an attractive representative set to investigate I/R injury since it is readily accessible to experimental manipulations in vivo and in vitro. Moreover, I/R injury underlie many retinal diseases such as, glaucoma, diabetic retinopathy, and central retinal artery occlusion [22], which are the leading causes of visual impairment or blindness. In our study, therefore, we used mouse model of retinal I/R injury to explore the molecular mechanisms and potential therapeutic targets. We identified lncRNA-H19 as a crucial player and uncovered its specific mechanism in retinal I/R, which can potentially serve as a therapeutic target. Mechanistically, lncRNA-H19 functioned through forming a ceRNET with miR-21 and PDCD4 to regulate NLRP3/6 inflammasome balance, pyroptosis and sterile inflammation. Furthermore, this ceRNET also directly modulate neuronal death and mitochondrial dysfunction. These findings provide novel insights into the mechanisms of I/R injury and facilitated prophylaxis and treatment of I/R-mediated diseases.

## Results

### Dysregulated transcriptomes in the I/R retina

**Fig. 1:**
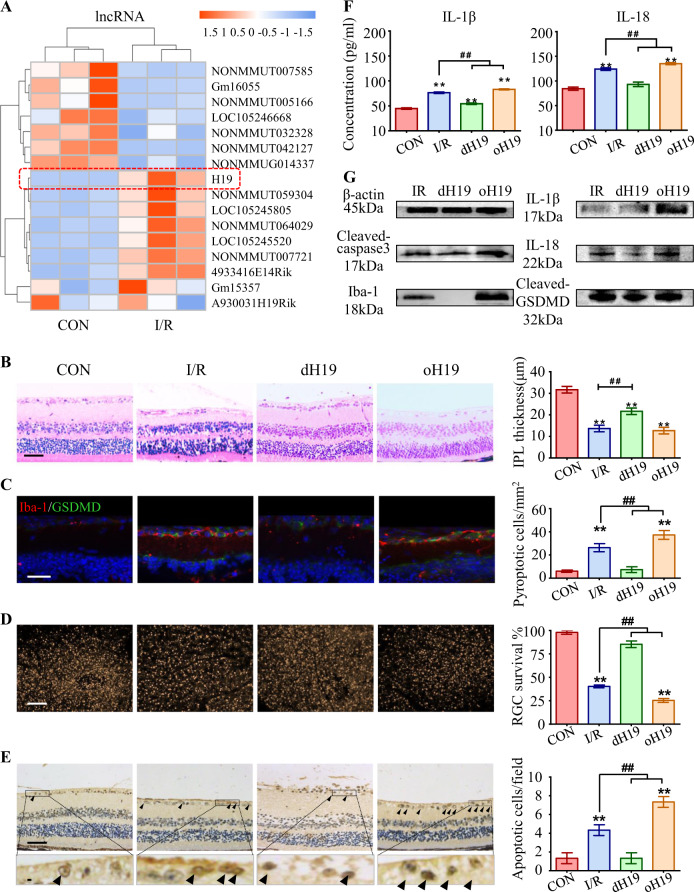
Predominant role of H19 in microglial pyroptosis and neuronal apoptosis. **a** Heat map listed the top 16 dysregulated lncRNAs in response to I/R injury. LncRNA H19 (red box) was the most upregulated lncRNA in the I/R retina. **b** Retinas exposed to I/R injury displayed a significant decrease in IPL thickness. This I/R-induced IPL attenuation was significantly ameliorated in dH19 mice and aggravated by H19 overexpression in retinas. **c** Iba-1+ microglia (red) had increased GSDMD-N (green) localized in the plasma membrane of I/R-treated mice compared with that of control retinas. Of note, less co-expression of Iba-1 and GSDMD-N was observed in the retina of dH19 mice. H19 overexpression promoted GSDMD-N augmentation in activated microglia (Iba-1+). **d** As measured by FG retro-labeling, viable RGC was presented as gold dots in flat-mounted retina. The RGC survival rate was noticeably decreased in response to I/R injury. H19 overexpression accentuated RGC apoptosis with less viable RGCs in retinas, which was prevented by H19 knockout. **e** Compared with the wild type counterparts, the dH19 retina had less TUNEL-positive cells (black arrows) in response to I/R injury, indicating that apoptosis was inhibited by H19 knockout. However, H19 overexpression markedly increased TUNEL-positive cells in GCL. **f** In retinal homogenates, H19 excision effectively inhibited the I/R-mediated overproduction of IL-1β and IL-18. This anti-inflammatory effect was abolished by H19 overexpression as measured by ELISA. **g** H19 knockout effectively prevented I/R-induced caspase-3 cleavage, indicating the pro-apoptotic effect of H19. Also, the retinas of H19-null mice exhibited lower protein levels of Iba-1, cleaved-GSDMD, IL-1β, and IL-18. The relative level of each target protein was normalized to β-actin from the same sample (Fig. S2F). Scale bar = 100 μm. Data were represented as means ± SD (*n* = 6). Compared with the normal control (CON): **P < 0.01. Compared with the I/R retina: ^##^*P* < 0.01. I/R, ischemia and reperfusion; dH19, H19 knockout; oH19, H19 overexpression; GCL, ganglion cell layer; IPL, inner plexiform layer; FG, flurogold

To identify dysregulated lncRNAs in the I/R retina, lncRNA/mRNA/miRNA composite arrays and bioinformatics analysis were recruited (see Methods). We found 618 significantly aberrant lncRNAs (*P* < 0.05 by the Wilcoxon signed-rank test) among I/R retinas. The top 16 differentially expressed lncRNAs are listed in the heat map (Fig. 1a). The Pearson's correlation coefficients between the same experiments were 0.9982/0.9982 (IR/NC) among three I/R retinas and normal control (NC) retinas, which ensured consistency.

To assign biological functions to dysregulated mRNAs in I/R retinas, Kyoto Encyclopedia of Genes and Genomes (KEGG) and Panther analysis were used. We found that canonical pathways related to neuro-inflammation and neuronal lesions were mainly involved (Fig. S1A, B).

As major performer in neuro-inflammation and neuronal lesion, microglia and RGCs were purified from I/R retinas (see Methods) to validate five candidate lncRNAs (Gm33675, H19, LOC105246304, 4930545L08Rik, and Gm30934) (Fig. S1C). Interestingly, H19 knockdown was capable of regulating excessive cytokine production and RGC viability during oxygen-glucose deprivation and reperfusion (OGD/R). However, Gm33675, LOC105246304, 4930545L08Rik, and Gm30934 siRNAs lacked efficacy in regulating RGC viability or cytokine production in supernatant of microglia cells (Fig. S1D, E). Further confirmed by real-time PCR, I/R significantly increased H19 levels in both RGCs and microglia, indicating a potential role of H19 in these two types of cells during I/R injury (Fig. S2A). In summary, H19 is a promising manipulator of I/R-induced sterile inflammation and neuronal lesions.

### Crucial roles of H19 in microglial pyroptosis and neuronal apoptosis

To evaluate the specific role of the lncRNA H19 in retinal I/R damage, H19 was knocked out or overexpressed in retinas before the onset of retinal ischemia (see Methods, Fig. S2C, D).

During I/R injury, RGCs dendrites were damaged first (attenuating the IPL thickness) and later cause the loss of RGC somas (decreasing cell number in GCL) [23]. Therefore, IPL thickness and cell density in GCL were quantified to measure the I/R-induced damage on neuronal death. Regarding these histological features, H19-deficient (dH19) and overexpressed (oH19) mice presented no significant difference in histological structures compared with wild-type C57 mice, especially in the thickness of the inner plexiform layer (IPL) and cell density of the ganglion cell layer (GCL) (Fig. S2E). However, retinas exposed to I/R displayed a significant decrease in IPL thickness (13.7 ± 0.9 μm) compared with normal retinas (31.7 ± 0.9 μm) 7 days after reperfusion. I/R-induced retinal damage, especially attenuation in IPL, was significantly ameliorated in dH19 mice (21.7 ± 0.8 μm) and aggravated by oH19 (12.7 ± 0.8 μm) (Fig. 1b). Consistently, surviving cells decreased in the GCL of retinas that underwent I/R injury (47.7 ± 2.3 cells/mm^2^ vs 86.3 ± 1.2 cells/mm^2^ in control retinas), which was relieved by dH19 (88.3 ± 2.1 cells/mm^2^) and augmented by oH19 (31.3 ± 1.3 cells/mm^2^) (Fig. S2B).

Iba-1 was used to label activated microglia [24]. I/R injury increased GSDMD-N expression in Iba-1+ microglia, implying I/R promoted microglial pyroptosis in the retina. In addition, H19 overexpression promoted I/R-induced pyroptosis with augmented GSDMD-N. Although there was also an increase of full length GSDMD in cytosol, the strong signal in plasma membrane, where the GSDMD-N specifically enriched to form pores, confirmed the typical role of pyroptosis in response to I/R and H19 overexpression. However, aberrant pyroptosis was inhibited by H19 knockout with lower co-expression of Iba-1 and GSDMD-N in microglia (Fig. 1c), thus exerted protection against I/R injury.

Moreover, flurogold retro-labeling was used to label the viable RGCs in the retinas as gold dots. As presented in the flat-mounted retinas, RGCs underwent noticeable apoptosis in response to I/R injury (59.7 ± 0.9% vs 2.0 ± 1.56% in normal control). H19 overexpression accentuated RGC apoptosis (74.7 ± 1.2%) with less gold dots, which was mostly prevented in H19-deficient retinas (14.7 ± 2.0%) (Fig. 1d). TUNEL staining further confirmed the anti-apoptotic role of H19 knockout (1.3 ± 0.3  cells vs 4.3 ± 0.3 TUNEL + cells in the I/R group) in response to I/R injury (Fig. 1e).

Meanwhile, accumulation of IL-1β and IL-18 further confirmed the pro-inflammatory role of H19 as measured by ELISA, which was prevented in dH19 retinas (Fig. 1f). Microglial pyroptosis and neuronal apoptosis were activated in I/R retinas at both transcriptional and translational levels, which was further promoted by oH19 and inhibited by dH19 (Fig. 1g, Fig. S2F, G). These findings suggest that H19 played a crucial role in I/R-induced retinal damage by regulating microglial pyroptosis and consequent RGC apoptosis.

### H19 regulated neuro-inflammation by rebalancing NLRP3/NLRP6 inflammasomes

**Fig. 2:**
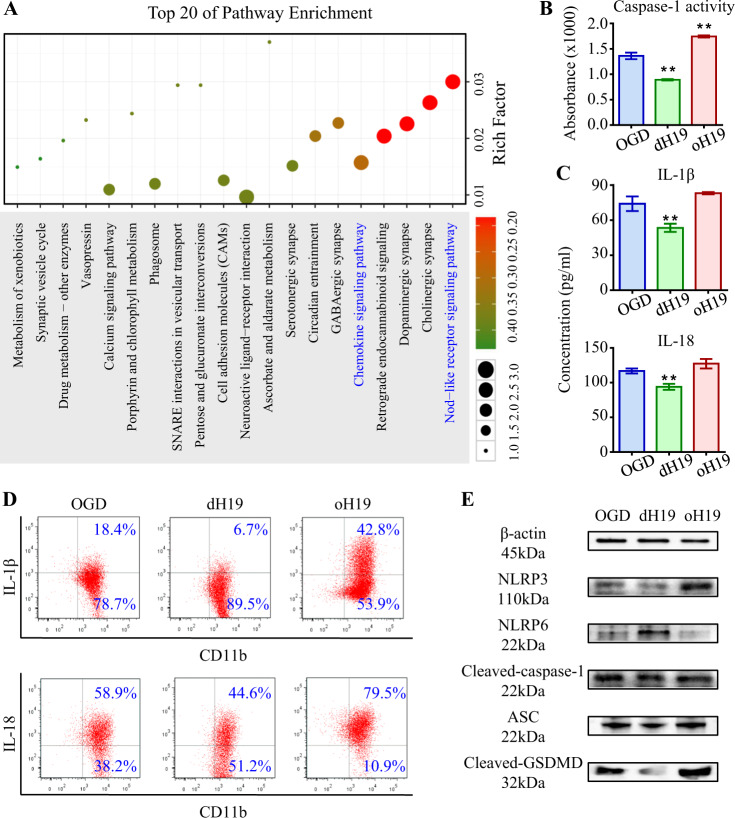
H19 regulates neuro-inflammation by rebalancing NLRP3/NLRP6 inflammasomes. **a** GO analysis annotated biological function to dysregulated mRNAs in I/R retina. Canonical pathways related to Nod-like receptor signaling and chemokine production acquired vital importance in I/R injury. **b** In primary microglia, H19 knockout significantly prevented OGD/R-induced caspase-1 activation, which was exacerbated by H19 overexpression. **c** As measured by ELISA, OGD/R-induced overproduction of IL-1β and IL-18 was suppressed by H19 knockout and aggravated by H19 overexpression in cultured microglia. **d** As measured by flow cytometry, H19 excision effectively decreased IL-1β or IL-18 overproduction in microglia. And H19 overexpression increased production of these two pro-inflammatory cytokines. **e** Reciprocal activation of NLRP3/NLRP6 inflammasomes was observed in microglia underwent OGD/R. Imbalance activation of inflammasomes resulted in increased ASC and cleavage of caspase-1 and GSDMD. The upregulation of these pro-inflammatory proteins was aggravated in oH19 microglia as attenuated by H19 knockout as measured by immunoblot. The relative level of each target protein was normalized to β-actin from the same sample (Fig. S2H). Data were represented as means ± SD (*n* = 6). Compared with the OGD/R group (OGD): **P < 0.01. OGD/R, oxygen-glucose deprivation and reperfusion; dH19, H19 knockout; oH19, H19 overexpression; ASC, apoptosis-associated speck-like protein containing a CARD

The sudden release of microglial contents during pyroptosis brought about severe inflammation in the entire retina. We then explored if H19 is capable of regulating neuro-inflammation, as well as its underlying mechanisms. To this end, Gene ontology (GO) analysis was used. We found a predominant role for the Nod-like receptor (NLR) and chemokine signals in I/R-induced retinal inflammation (Fig. 2a). NLR proteins (NLRP) have been shown to be critical sensors of damage and contributors of pyroptosis [25]. Based on these, we supposed that H19 regulated retinal inflammation via NLRP inflammasomes.

OGD/R significantly activated caspase-1, the executioners of NLR-related inflammasomes. H19 knockout effectively inhibited caspase-1 activity, which was augmented by H19 overexpression in microglia (Fig. 2b). Consistently, the maturation and release of inflammatory cytokines, which was facilitated by activated caspase-1, was increased by OGD/R. Furthermore, IL-1β and IL-18 production was increased by oH19 as measured by flow cytometry and ELISA. On the contrary, H19 knockout exerted anti-inflammatory effects by inhibiting the production of these cytokines (Fig. 2c, d).

We also observed significantly increased production of NLRP3 mRNA and protein, a reciprocal response in contrast to the NLRP6 inflammasome, which was aggravated in oH19 microglia as attenuated by dH19 (Fig. 2e, Fig. S2H, I). Reciprocally activated NLRP3 and NLRP6 inflammasomes triggered activation of caspase-1, which resulted in the accumulation of IL-1β and IL-18 as stated above. In conclusion, H19 regulated neuro-inflammation by resetting the balance between NLRP3/NLRP6 inflammasomes.

### H19 sponged miR-21 to regulate neuro-inflammation

**Fig. 3:**
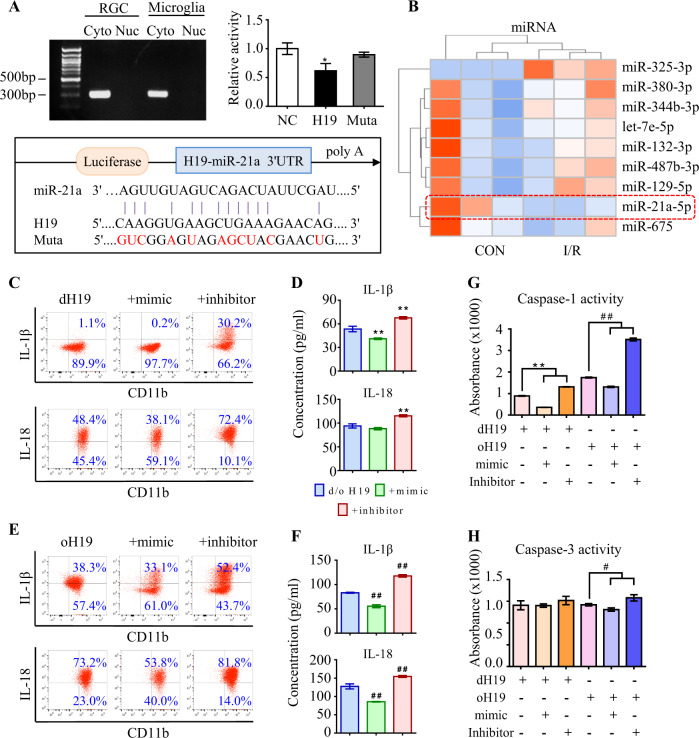
H19 sponges miR-21 to regulate neuro-inflammation. **a** As measured by qPCR, H19 was specifically expressed in the cytoplasm instead of the nucleus in both RGCs and microglia. Compared to NC RNA, miR-21 reduced luciferase activity by 43.4% ± 1.4% through binding to H19. The miR-21-dependent inhibition of luciferase activity was abolished after mutations (red) in the binding site. Data were represented as means ± SD (*n* = 3). Compared with NC RNA: *P < 0.05. **b** The top nine dysregulated miRNAs in I/R retinas. **c,** **d** Anti-inflammatory effect of H19 excision was further promoted by the miR-21 mimic with less IL-1β and IL-18 production in supernatant of retinal microglia. As measured by flow cytometry and ELISA, the miR-21 inhibitor greatly antagonized the anti-inflammation effect of dH19 with more production of IL-1β and IL-18. **e,** **f** In H19 overexpressed microglia, miR-21 downregulation further aggravated the production of IL-1β and IL-18, which was ameliorated by miR-21 upregulation as measured by flow cytometry and ELISA. **g,** **h** Caspase-1 activity was further restrained in the microglia deficient of both H19 and miR-21 compared with the single H19 excision. The miR-21 inhibitor further augmented caspase-1 activity in oH19 microglia. Either H19 or miR-21 had no significant effect on apoptotic activity of caspase-3 in microglia. Data were represented as means ± SD (*n* = 6). Compared with the dH19 group: **P < 0.01. Compared with the oH19 group: ^##^*P* < 0.01. Cyto, cytoplasm; Nuc, nucleus; NC, normal control; Muta, mutation; dH19, H19 knockout; oH19, H19 overexpression

As measured by fluorescence in situ hybridization (FISH), H19 was mainly distributed in the cytoplasm instead of nucleus among retinal cells (Fig. S3A). More importantly, I/R injury dramatically upregulated H19 expression in both RGCs and microglia compared with normal control (Fig. S2A). In consistent, H19 was found predominantly localized in the cytoplasm by qPCR analysis (Fig. 3a), endowing its potential function as a competing endogenous RNA (ceRNA) to sequester miRNAs. To identify the potential targets of H19, dysregulated miRNAs in response to I/R injury were analyzed (Fig. 3b). We examined miR-675-3p (miR-675) and miR-21a-5p (miR-21), the putative targets of H19 by luciferase report assay (see Methods). Compared to control non-coding RNA (ncRNA), miR-21 reduced luciferase activity by 43.4% ± 1.4% through binding to a target sequence. Furthermore, miR-21-dependent inhibition of luciferase activity was abolished after mutations in the H19 binding site (Fig. 3a). By contrast, miR-675 had little influence on luciferase activity (Fig. S3B, *P* > 0.1). Moreover, a negative correlation between miR-21 and H19 was demonstrated by detecting comparable copy numbers of H19 and miR-21 in RGCs and microglia (Fig. S3C, D).

MiR-21 was found to effectively resume the NLRP3/6 inflammasome balance and inhibit production of pro-inflammatory cytokines. Moreover, the miR-21 mimic dampened the cleavage of caspase-1 and GSDMD, thus suppressing OGD/R-induced pyroptosis in primary microglia (Fig. S4A–E).

Thereafter, we wondered if H19 could regulate neuro-inflammation through sponging miR-21. To this end, the expression levels of H19 and miR-21 were co-regulated before the onset of OGD/R treatment. In dH19 microglia, miR-21 upregulation further enhanced the protective effect by inhibiting IL-1β (1.05 ± 0.08% to 0.15 ± 0.05%) and IL-18 (48.5 ± 0.12% to 38.0 ± 0.11%) production (Fig. 3c, d). By contrast, dH19-mediated anti-inflammation was significantly destroyed by the miR-21 inhibitor (Fig. 3e, f). The miR-21 mimic consistently restrained activation of caspase-1 but not caspase-3 in dH19 microglia, implying that H19 and miR-21 worked collaboratively in I/R-induced pyroptosis (Fig. 3g, h). Mechanistically, the miR-21 inhibitor augmented the NLRP3/6 imbalance and consequent neuro-inflammation in both dH19 and oH19 microglia. The miR-21 mimic rescued the pro-inflammatory effect of oH19 by rebalancing NLRP3/6 inflammasomes and preventing microglial pyroptosis (Fig. S5A–F). These results confirmed that H19 regulates neuro-inflammation through sponging miR-21 in vitro.

### H19 competed with PDCD4 for miR-21 to form the ceRNET

**Fig. 4:**
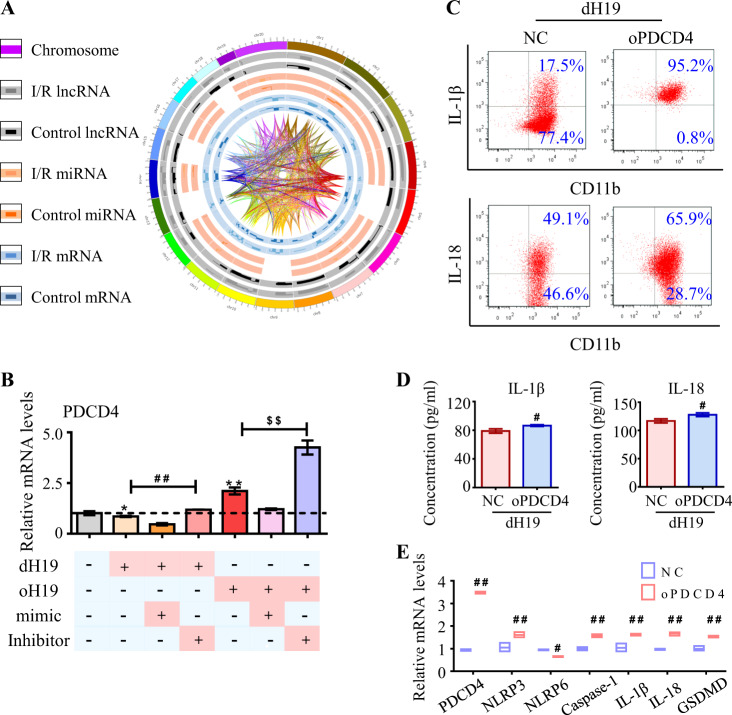
H19 competed with PDCD4 for miR-21 to form ceRNET. **a** Sketch of the dysregulated transcriptome and their correlations. From outside in: chromosome position, lncRNAs in the I/R retina, lncRNAs in the healthy control, miRNAs in the I/R group, miRNAs in the control group, mRNAs in the I/R retina, mRNAs in the healthy control, and interactions among these three types of RNAs in the dysregulated transcriptome. **b** In cultured microglia, PDCD4 expression was up-regulated by oH19 and the miR-21 inhibitor, and down-regulated by dH19 and the miR-21 mimic. PDCD4 dysregulation was aggravated by simultaneous H19 and miR-21 treatment at transcriptional level. **c,** **d** As measured by flow cytometry and ELISA, H19 excision-mediated prevention of IL-1β and IL-18 overproduction was abolished by PDCD4 overexpression in microglia. **e** As measured by real-time PCR, PDCD4 competed with H19 in regulating the NLRP3/NLRP6 inflammasome balance in microglia. And the expression of caspase-1, GSDMD, and the pro-inflammatory cytokines was increased by PDCD4 overexpression. Data were represented as means ± SD (*n* = 6). Compared with the OGD/R group: *P < 0.05, **P < 0.01. Compared with dH19 microglia: ^#^*P* < 0.05, ^##^*P* < 0.01. Compared with oH19 microglia: ^$$^*P* < 0.01. NC, normal control lentivirus; dH19, H19 knockout; oH19, H19 overexpression; oPDCD4, PDCD4 overexpression

We then further illustrated how the non-coding components, H19 and miR-21, associated with coding genes to construct the ceRNET. Assisted by interrelated analysis in the dysregulated transcriptome, we found several candidate genes (Fig. 4a). Among these, PDCD4 was nominated as an important target by sharing the same response elements between miR-21 and H19 [9]. Luciferase reporter assay was recruited to test the direct binding between miR-21 and PDCD4. As showed in Fig. S6A, the combination of miR-21 mimic to PDCD4 greatly decreased the luciferase activity. Furthermore, mutation in their binding site inhibited the decrease of luciferase activity, indicating that PDCD4 is the target gene of miR-21.

In addition, PDCD4 decreased in response to either H19 knockout or miR-21 upregulation as measured in mRNA and protein levels. By contrast, oH19 and miR-21 inhibitors significantly increased PDCD4 expression, which was further augmented by oH19 together with the miR-21 inhibitor (Fig. 4b, Fig. S6B, C). These results confirmed the essential role of PDCD4 in ceRNET.

As the competitor of H19 in ceRNET, PDCD4 overexpression almost completely blocked the anti-inflammatory effects of dH19 in microglia as measured by both flow cytometry (IL-1β 95 ± 0.7% vs 17.3 ± 0.6% in the dH19 group, IL-18 65.2 ± 0.7% vs 49.7 ± 0.9% in the dH19 group), and ELISA (IL-1β 91 ± 1 pg/ml vs 78 ± 2 pg/ml in the dH19 group, IL-18 128 ± 2 pg/ml vs 117 ± 2 pg/ml in the dH19 group) (Fig. 4c, d). These all attributed to PDCD4-activated NLRP3 inflammasome and suppressed NLRP6 in microglia. Meanwhile, activated caspase-1 cleaved GSDMD to exert perforated cytotoxity (Fig. 4e, Fig. S6D). Collectively, these findings indicated that H19 competitively bound with miR-21 to facilitate PDCD4 expression in ceRNET, thus triggering neuro-inflammation.

### H19/miR-21/PDCD4 ceRNET directly regulated RGC apoptosis via caspase signaling

I/R injury not only increased H19 expression in microglia but also in RGCs, indicating the potential role of H19 in I/R-induced RGC death. As reported, both I/R injury itself and pyroptosis-mediated sterile inflammation lead to neuronal apoptosis, which resulted in the irreversible loss of retinal function [26]. Therefore, we then assessed if the H19/miR-21/PDCD4 ceRNET also participates in regulating neuronal apoptosis. OGD/R-induced RGC apoptosis was inhibited by dH19 (40.4 ± 0.4% vs. 50.1 ± 0.1% in the OGD group) and aggravated by oH19 (89.2 ± 0.4% vs. 49.9 ± 0.2% in control lentivirus transfected cells) as measured by flow cytometry (Fig. S7A). MiR-21 was also found to prevent RGC apoptosis in vitro. Activation of apoptotic caspases, including caspase-8 and caspase-3, was consistently suppressed by H19 excision and miR-21 upregulation (Fig. S7A, B).

Furthermore, the neuroprotective effect of dH19 was significantly but incompletely reversed by the miR-21 inhibitor (67.5 ± 0.8% surviving RGCs) or PDCD4 overexpression (63.4 ± 1.1% surviving RGCs). By contrast, the disruption in caspase-mediated apoptotic signaling and RGC death during H19 overexpression (89.7 ± 3.5%) was ameliorated by the miR-21 mimic (48.8 ± 2.1%) (Fig. S8A–C). Real-time PCR and western blot results further confirmed the regulatory effect of the ceRNET in OGD/R-induced neuronal apoptosis (Fig. S7, S8). Collectively, these findings indicated that the H19/miR-21/PDCD4 ceRNET directly regulated RGC apoptosis via caspase-mediated apoptotic signaling.

### H19/miR-21/PDCD4 ceRNET induced mitochondrial dysfunction in I/R damage

Mitochondrial membrane potential (MMP) collapse and reactive oxygen species (ROS) overproduction have been recognized as alarm signals that trigger NLRP3 activation and pyroptosis [27, 28]. This information prompted us to investigate if mitochondrial dysfunction was involved in I/R-induced retinal damage.

OGD/R greatly hindered MMP (marked by an intracellular fluorescence shift from red to green) in microglia. This MMP collapse was rescued by H19 excision and augmented by H19 overexpression (Fig. S9A). Meanwhile, oH19 promoted intracellular ROS accumulation as measured by flow cytometry and fluorescent assay (Fig. S9B). The miR-21 mimic suppressed MMP collapse and ROS accumulation, which was reversed by the miR-21 inhibitor in primary microglia (Fig. S9C, D).

Working in the ceRNET, H19 overexpression decreased MMP, which was further aggravated by the miR-21 inhibitor. PDCD4 overexpression almost completely blocked the mitochondrial protective effects of dH19 on microglia exposed to OGD/R with regard to MMP collapse and intracellular ROS accumulation (FITC+). However, dH19 treatment together with the miR-21 mimic observably protected mitochondrial function by restoring MMP and preventing OGD/R-induced ROS overproduction (Fig. S10A–F).

In conclusion, the H19/miR-21/PDCD4 ceRNET directly protected mitochondrial function in I/R damage.

### H19 functioned in the ceRNET during I/R-induced retinal damage

We further investigated the role of the H19/miR-21/PDCD4 ceRNET in neuro-inflammation and neuronal apoptosis in vivo. To this end, H19, miR-21, and PDCD4 were simultaneously knocked out or overexpressed in mice retinas before I/R (see Methods, Fig. S11A–C).

**Fig. 5:**
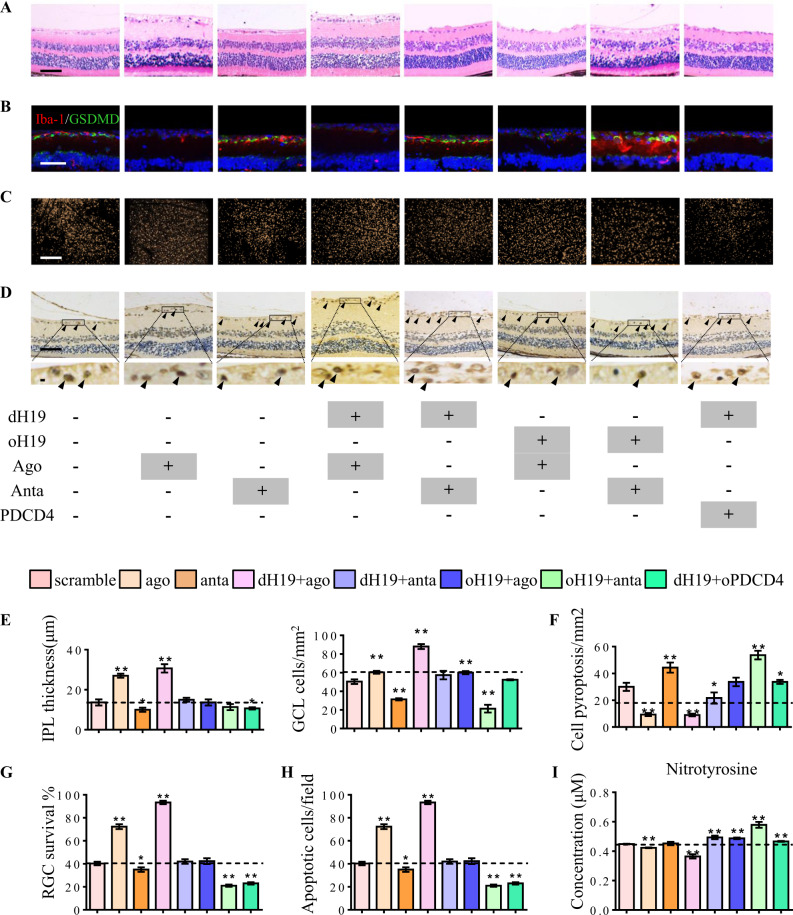
H19 functions in ceRNET during I/R-induced retinal damage. **a,** **e** Neuroprotective effect of H19 excision was enhanced by miR-21 up-regulation via inhibiting IPL attenuation and cell death in GCL. miR-21 downregulation or oPDCD4 eliminated the protective effect of H19 knockout. H19 overexpression and miR-21 antagomir worked both singly and together to increase I/R-induced retinal damage by augmenting IPL attenuation and cell death in GCL. **b**, **f** Microglial pyroptosis was suppressed by dH19 and miR-21 upregulation alone or together as shown by less cells co-labeled with Iba-1 (red) and GSDMD-N (green). H19 overexpression, miR-21 knockdown, and oPDCD4 augmented I/R-induced microglial pyroptosis with more GSDMD-N anchored in the plasma membrane of Iba-1+ cells. **c**, **g** H19/miR-21/PDCD4 worked in ceRNET to regulate RGC survival (gold dots) in I/R retinas. Both H19 knockout and miR-21 upregulation protected RGCs from I/R-induced apoptosis. By contrast, H19 overexpression and PDCD4 increased RGC apoptosis in response to I/R injury as shown by fewer gold dots in retinal flat mounts. **d**, **h** Compared with the wild type retina, dH19 excision and miR-21 upregulation exhibited fewer TUNEL-positive cells (black arrows) in response to I/R injury. This protective effect was eliminated by H19 overexpression, miR-21 down-regulation, or PDCD4 with markedly increased TUNEL+ cells in GCL. **i** Nitrotyrosine (ROS derivative) accumulation in retinas was significantly decreased by dH19 and miR-21 agomir. H19 overexpression, miR-21 antagomir, and oPDCD4 increased nitrotyrosine accumulation in response to I/R. Scale bar = 100 μm. Data were represented as means ± SD (*n* = 6). Compared with the I/R group: *P < 0.05, **P < 0.01. dH19, H19 knockout; oH19, H19 overexpression; oPDCD4, PDCD4 overexpression; ago, agomir of miR-21; anta, antagomir of miR-21

Our results showed that H19 excision inhibited IPL attenuation and cell loss in GCL. This protective effect was promoted by miR-21 and abolished by PDCD4 (Fig. 5a, e). Moreover, H19 overexpression increased microglial pyroptosis in response to I/R injury. Aberrant pyroptosis was further augmented by miR-21 downregulation with elevated levels of GSDMD in Iba-1+ microglia (Fig. 5b, f). This pro-inflammatory effect was also confirmed by ELISA, which showed increased IL-1β and IL-18 production with oH19 and miR-21 anta treatment (Fig. S11D). As measured by flurogold retro-labeling and TUNEL assay, RGC apoptosis was also directly regulated by the H19/miR-21/PDCD4 ceRNET (Fig. 5c, d, g, h). Decreased nitrotyrosine (a protein derivative of ROS) accumulation in the retina was also involved in the ceRNET-exerted protection (Fig. 5i).

Mechanistically, imbalanced activation of NLRP3/NLRP6 inflammasomes was effectively prevented by dH19 together with miR-21 upregulation as detected in transcriptional and translational levels. Consistently, the cleavage of caspase-1 and GSDMD was also inhibited by H19 excision and miR-21 upregulation. Meanwhile, apoptotic signaling was disrupted by H19 overexpression together with decreased miR-21. PDCD4 almost completely blocked the anti-inflammatory and neuroprotective effect of dH19 in the retina (Fig. S11E, Fig. S12A–C). These results indicated that the H19/miR-21/PDCD4 ceRNET exerted pivotal regulatory effects in both neuro-inflammation and neuroprotection in mice retinas during I/R.

## Discussion

The absence of the damage initiator, as well as validated therapeutic targets, has hindered the development of an effective therapy for retinal I/R injury. This study has four main findings and implications. First, we demonstrated lncRNA H19 as a crucial onset in I/R diseases. Second, we reported the identification and functional characterization of H19-leaded ceRNET, which expands our understanding of I/R damage. Third, the importance of inflammasome balance provided a novel direction for understanding and treating immunological-related diseases. Fourth, microglial pyroptosis was confirmed to be pivotal in I/R-induced sterile inflammation.

**Fig. 6:**
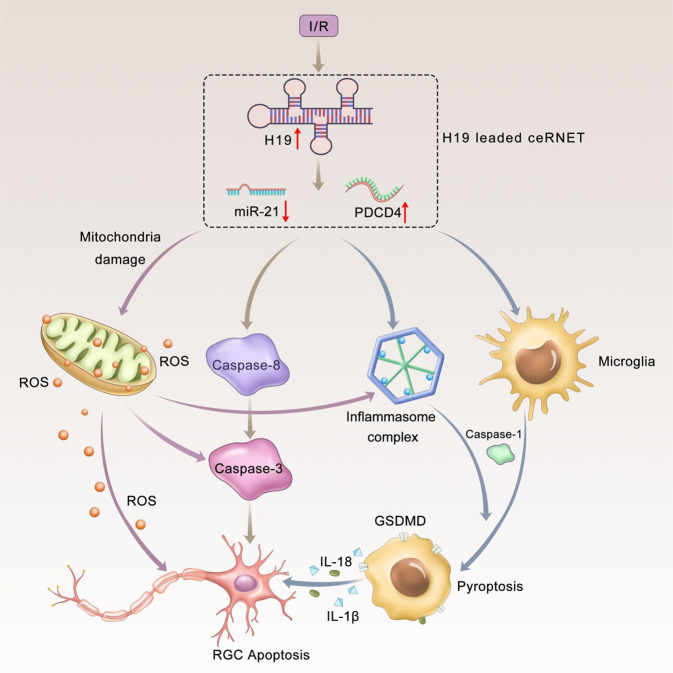
Graphical summary of H19/miR-21/PDCD4 ceRNET in I/R injury. H19 responded to ischemic insults with increased expression level. Upregulated H19 then competed with PDCD4 for miR-21 to form the ceRNET. The ceRNET is capable of processing several pathological signals into a unitary and coherent adaptive response. H19-mediated ceRNET triggered reciprocal activation of NLRP3/NLRP6 inflammasomes. Thereafter, activated caspase-1 was recruited to cleave GSDMD, which characterized microglial pyroptosis. Meanwhile, on the inflammasome platform, mature forms of IL-1β and IL-18 were managed, which mediated pyroptosis to exert neurotoxicity. Besides inflammatory caspases, the H19/miR-21/PDCD4 ceRNET also activated apoptotic caspases (caspase-3 and caspase-8) to directly regulate RGC apoptosis in I/R injury. Mitochondrial dysfunction (marked by MMP collapse) and consequent ROS overproduction were identified as independent activators of inflammasomes and apoptotic signals. H19 knockout effectively relieved I/R-induced neuro-inflammation, neuronal death and mitochondrial failure. Simultaneous regulation of the H19/miR-21/PDCD4 ceRNET further blocked the ischemic cascade to improve the protective effect. I/R, ischemia/reperfusion injury; RGC, retinal ganglion cell; MMP, mitochondrial membrane potential; ROS, reactive oxygen species

H19 is one of the most highly expressed and conserved transcripts in mammalian development [29]. We have demonstrated the pivotal role of H19 among the ischemic cascade in regulating sterile inflammation and neuronal apoptosis. Mechanistically, H19 increased in response to ischemia insult and sponged miR-21 to facilitate PDCD4 expression. H19 reciprocally activated NLRP3/NLRP6 inflammasomes to initiate GSDMD cleavage and microglial pyroptosis. In addition to the increased cleavage in caspase-1 and GSDMD, we also witnessed the increase of mRNA levels of these two genes in response to H19 overexpression (Fig. 4e, S2I, S3H, S4 C&F, and S10E). These effects may rely in the transcriptional regulation ability of H19. H19 was found to regulate the mRNA transcription via recruiting more transcription factor [30], including Foxo1 [31] and E2F [32]. Also, H19 was capable of promoting phorsphorylation and nuclear transmition of transcription factors [33], thus increasing the expression of target genes. Then, more cleaved caspase-1 is recruited to produce mature forms of IL-1β and IL-18 and trigger sterile inflammation. Meanwhile, H19 activated caspase-8 and caspase-3 to induce RGC apoptosis. Mitochondrial dysfunction was also involved to trigger the activation of the inflammasome complex and caspase-mediated apoptotic signal (Fig. 6). Consistently, H19 excision effectively relived I/R-induced sterile inflammation, neuronal death, and mitochondrial failure. Therefore, as a central hub in ischemic pathogenesis, H19 is likely to be a promising therapeutic target with great efficacy, because it can synchronously regulate multiple detrimental signals. Moreover, H19 can kill several “bad birds” with one stone through the regional delivery of siRNA or gene editing. Thus, we can avoid side effects from drugs targeting multiple participating mechanisms. Our study also provided a novel avenue for developing therapeutics, i.e., excavating the central hub in pathogenesis, which facilitates the prophylaxis and treatment of other complex diseases.

Besides I/R-induced apoptosis, recent findings showed that neurons may also suffer from pyroptotic death [34]. As reported, neuronal caspase-1 activation was found in hypoxia/ischemia and traumatic injured neuron cultures [35]. Pharmacological inhibition of caspase-1 activity protected neuronal death induced by brain ischemia or trophic factor withdrawal [36, 37]. Also, knockdown of NLRP1 or caspase-1 reduced neuronal loss in animal models of temporal lobe epilepsy [38] and Alzheimer’s disease [39]. However, inhibition of inflammasome or caspase-1 also blocks neuro-inflammation so that it is not clear whether neuronal death is induced by inflammation or pyroptosis (or both). To distinguish between these possibilities, we can measure the level of GSDMD, especially the N-terminal in purified neuron cultures. Moreover, the typical pore formation on plasma membrane can be a specific marker in our future researches.

Our results supported the contention that ceRNET uncovers the regulatory networks that have been overlooked by conventional protein-coding-centered studies, for two main advantages. First, ceRNET responds at an earlier stage to damage. Transcriptional regulation reacts more quickly than translational regulation by triggering RNA expression. Second, ceRNET represents a robust platform for transcripts on the basis of shared miRNAs [40]. Therefore, ceRNET could functionalize non-coding transcripts in normal development and pathological conditions, such as in the control of muscle differentiation [41], epithelial-to-mesenchymal transition [42], and cancer progression [43]. We believe that ceRNET will further illustrate disease processes and present opportunities for new therapies.

Particularly worth mentioning is that our finding regarding the balance between NLRP3 and NLRP6 inflammasomes can enhance our understanding of I/R injury. Recent studies supposed that the NLRP3 inflammasome in glial cells can detect cellular damage and mediate inflammatory responses during CNS, myocardial, renal, and hepatic I/R injury [16, 44]. By contrast, the NLRP6 inflammasome is a critical suppressor of cytokine production [45], which can protect against intracerebral hemorrhage [46] and intestinal inflammation [47]. The expression of inflammasome-associated proteins, such as NLRP3, NLRP6, ASC, and caspase-1, were examined in this study to assess inflammasome activity. Our findings indicated that the increased activity of the NLRP3 inflammasome and IL-1β and IL-18 positively associated with structural and functional damage in the retina. The activity and protein levels of the NLRP6 inflammasome were negatively correlated with IPL attenuation, GCL cell loss, and RGC apoptosis. Our results indicated a contrasting regulation between NLRP3 and NLRP6 inflammasomes in retina I/R injury, which suggests that pharmacologically or transcriptionally targeting the balance may inhibit tissue-disruptive events and prevent I/R-related diseases. This evidence can serve as an attractive alternative for understanding and treating other immunological-related diseases.

In summary, we found that lncRNA H19 can serve as a crucial manipulator and therapeutic target in retinal I/R injury, by simultaneously regulating sterile inflammation and neuronal apoptosis. ceRNET, inflammasome balance and pyroptosis were introduced to I/R injury for the first time, perfecting the pathological sketch of I/R-related diseases. More broadly, our work provided new insights in developing versatile therapeutic targets, which may facilitate prophylaxis and treatment of I/R injury.

## Methods

### Animals

Six-week-old C57BL/6J male mice were purchased from the Guangdong Medical Laboratory Animal Center. H19 knockout (H19 ^−/−^) male mice (dH19, purchased from Viewsolid Biotech, CHN) were produced on the C57BL/6J background. The mice were fed and maintained according to the Association for Research in Vision and Ophthalmology Statement for the Use of Animals in Ophthalmic and Vision Research.

### Establishment of the retinal I/R model

The retinal I/R model were established as previously described [48]. In brief, a 30-gauge needle containing a balanced salt solution was cannulated into the anterior chamber to maintain the IOP at 70 mmHg for 60 min. The sham operation, which served as the control, was performed without elevating the IOP. After 60 min, the IOP was normalized by carefully withdrawing the needle. To prevent a bacterial infection, tobramycin ointment (Alcon, USA) was used.

In H19 or PDCD4 overexpressed mice, an adeno-associated virus (AAV) solution (1 μL, 10^12^ v.g/ml) loaded with specific targets (HANBIO, CHN) was delivered into the vitreous body four weeks before the onset of reperfusion to maximize the transfection efficiency. An antagomir/agomir solution containing microRNA was intravitreally injected (1 μL, 20 μM) before the onset of reperfusion. The normal control group received the vehicle (1 μL).

### Microarray profiling

Total RNA was isolated from the retina of I/R mice (*n* = 3) or age-matched and sex-matched normal control mice (*n* = 3) using TRIzol Reagent (Invitrogen, USA).

Microarray profiling was performed using the Agilent Mouse lncRNA+ mRNA Array V1.0 and the Agilent Mouse miRNA Microarray (Release 21.0, 8x60K). To select differentially expressed RNAs, we chose threshold values having a fold change of ≥2 and ≤−2 and a Benjamini–Hochberg corrected *P*-value of 0.05. KEGG biological system and Panther analysis were used to assign biological meaning to affected RNAs in response to retinal I/R damage.

### RGC labeling and survival quantification

In vivo RGC retrograde labeling was performed as previously described [48]. Bilateral holes were drilled, and 1 µL of a 4% (vol/vol) Fluorogold (FG, hydroxystilbamidine; Fluorochrome, USA) solution was injected into both superior colliculi. After the injection, the micro-syringe was unmoved for 30 s and then slowly removed. Finally, the dissected scalp was sutured, followed by the topical application of tobramycin.

To ensure maximal RGC labeling, animals were kept alive for 7 days to allow FG retrograde transport before sacrifice. FG-positive RGCs were identified in retinal flat mounts with an AxioImager fluorescent microscope (Carl Zeiss MicroImaging Inc., GER). Surviving RGCs were manually counted using ImageJ Software (LOCI, University of Wisconsin, USA). Six independent experiments were performed.

### Histological examination

Seven days after I/R damage, mice were overdosed with anesthesia and transcardially perfused with isotonic saline and 4% (wt/vol) paraformaldehyde (PFA). Mouse eyes were enucleated and stained with hematoxylin and eosin (H&E) [48]. To quantify retinal damage, the thickness of the inner plexiform layer (IPL) was measured within 1 mm of the optic nerve center using Axiovision Software (Carl Zeiss).

An immunofluorescent assay was carried out as previously described [49]. Images were captured with a Zeiss LSM 510 confocal laser scanning microscope (Carl Zeiss) and processed by Adobe Photoshop CS8. Table S1 lists the antibodies used in this study. Data from three sections per eye were averaged.

### Fluorescence in situ hybridization (FISH)

Retinal sections were deparaffinized, heat-treated, and digested with protease K. H19 probe was constructed and labeled with Cy5 by the Ribobio Technology Company (Guangzhou, China). The fluorescence signals were visualized and captured using a Zeiss LSM 510 confocal laser scanning microscope (Carl Zeiss). Raw images were processed using Photoshop v.7.1 (Adobe Systems Inc., San Jose, CA, USA).

### Quantitation of gene overexpression in the retina

Retinas were dissected and stained as whole-mounts and slices after enucleation. To examine the efficacy of the lentivirus in vivo, a GFP-labeled lentivirus was used. Brn-3a and CD11b antibodies were also used to selectively label RGCs and microglia, respectively. ImageJ Software was used to count cells from eight image fields per retina (200×, Carl Zeiss) in flat mounts. Data from three sections per eye were averaged.

### Isolation of primary retinal microglia

Retinal microglia were isolated from the retina of neonatal wild-type and H19 knockout C57BL/6J mice as previously described with minor modifications [50]. In brief, mixed glial cultures were prepared from the retina, followed by mechanical and chemical dissociations (papain containing 180 units/mL DNase, Sigma) were performed at 38 °C for 10–20 min. Retinal cells were resuspended in DMEM/F12 (Gibco, USA) supplemented with 10% (vol/vol) FBS, 100 U/ml penicillin, and 100 mg/ml penicillin/streptomycin(Gibco) and cultured for 2–3 weeks until reaching confluency. Detached microglia were collected by shaking flasks at 200 rpm in an orbital shaker for 6 h.

### Primary culture of retinal ganglion cells

Primary RGCs were isolated according to the protocol of Winzeler and Wang [51]. In brief, retinal cells were isolated from the retina of wild-type and dH19 mice and transferred into anti-mouse macrophage antibody-coated flasks (Cedarlane & Jackson Immuno Research, USA) to remove adherent macrophages. Non-adherent cells were transferred to Thy1.2 monoclonal antibody-coated flasks (Millipore Chemicon, USA) to collect adherent cells. Adherent RGCs were incubated in growth medium containing factors as previously described at 37 °C in 5% (vol/vol) CO_2_.

### Cell treatment

To maximally simulate interactions between different retinal cells, microglia were co-cultured with primary RGCs in transwell chambers. Cells were starved for 24 h before various treatments.

Delivery of the RNA reagent. Cells at 70% confluency were used for transfection. siRNA or the miRNA mimic/inhibitor was mixed with the transfection buffer and reagent complexes (Ribobio, CHN) according to the manufacturer’s instructions to a final concentration of 100 nM. A maximal transfection efficiency required at least 24 h of transfection.

Gene overexpression. A specific lentivirus (LV, HANBIO., CHN) was added into the cell culture medium at a multiplicity of infection (MOI) of 30 for 6–8 h. The transfection efficiency was guaranteed by puromycin (5 μg/ml, Solarbio, CHN) selection for 48 h.

### Establishment of the OGD/R model

The OGD/R model was established by replacing the culture medium with glucose-free DMEM (Gibco) after washing cells twice with PBS (Gibco). Cells were then placed in a modular incubator chamber (Billups-Rothenberg, Inc., USA) containing 5% (vol/vol) CO_2_ and 95% (vol/vol) N_2_ at 37 °C for 3 h. Control cells were incubated in serum-free medium supplemented with 4.5 g/L D-glucose under normoxic conditions (5% (vol/vol) CO_2_ and 95% (vol/vol) air) for the same duration. At the end of the exposure period, cells were returned to normoxic conditions and incubated with serum-free medium supplemented with glucose for 12 h. Six independent experiments were performed.

### Luciferase reporter assay

H19 transcripts (NR_130974.1) and PDCD4 mRNA with or without mutations within miR-21 binding sites were cloned into upstream of the luciferase reporter. The pGL-3 Basic Luciferase Vector (Promega, USA) lacking the H19 or PDCD4 transcript inserts served as the control. The renilla reporter pRL (Promega) plasmid was used to normalize the transfection efficiency. Data were expressed as relative light units for luciferase normalized to renilla luciferase activity. Three independent experiments were performed.

### Quantitative real-time PCR and qPCR

Total RNA was extracted from the retina and cultured cells with TRIzol Reagent (Invitrogen) according to the manufacturer’s instructions. Nuclear and cytoplasmic RNA was isolated from cells according to the protocol of Arash [52]. cDNA synthesis was carried out with the PrimeScript RT Master Mix (TaKaRa, CHN). The amplification reaction was performed with 25 cycles for β-actin and 30–32 cycles for the other transcripts. To determine relative expression, PCR products were run on 2% (wt/vol) agarose gels. Quantitative analysis was performed with the Light Cycler 480 Real-Time PCR System (Roche Molecular Systems, Inc., SUR). The expression of target mRNAs was measured and normalized to β-actin. Table S2 lists the primer sequences that were used in this study. Six independent experiments were performed.

### Western blot

Total protein was isolated from the retina or cultured cells and run on 10% (wt/vol) polyacrylamide gels following a standard protocol. The expression of target proteins was normalized to β-actin obtained from the same sample (taken as 1.0) and then quantified using ImageJ Software. Table S1 lists the primary antibodies that were used in this study. Six independent experiments were performed.

### Caspase activity assay

Caspase activity in retinal lysates and cells was measured using the CaspGLOW™ Fluorescein Active Caspase-1, 3, and 8 Staining Kit (BioVision, USA) according to the manufacturer’s instructions. Caspase activity was presented as the fluorescent intensity measured at Ex/Em = 485/535 nm. Six independent experiments were performed.

### Viability and apoptosis assays in RGCs

Flow cytometry was used to measure OGD/R-induced apoptosis of RGCs using a Propidium Iodide (PI)/Annexin V-FITC Detection Kit and an Annexin V-PE/7-AAD Detection Kit (BD Biosciences, USA) according to the manufacturer’s instructions. Flow cytometric analysis using FlowJo 7.6.2 (FlowJo, LLC) was performed following standard protocols. Annexin V^+^ cells were identified as apoptotic ones. Ten thousand cells were collected and analyzed in each flow cytometric assay. Six independent experiments were performed.

### Analysis of the MMP by JC-1 staining

Changes in the mitochondrial membrane potential (MMP) were explored using the fluorescent probe 5,5′,6,6′-tetrachloro-1,1′,3,3′-tetraethyl benzimidazolyl carbocyanine iodide (JC-1, Sigma, USA) according to the manufacturer’s instructions and then recorded using an inverted fluorescent microscope (Carl Zeiss) or flow cytometry. Ten thousand cells were collected and analyzed in each flow cytometric assay. Averaged results of six independent experiments were obtained for each condition.

### Detection of the intracellular ROS level

Accumulated intracellular reactive oxygen species (ROS) were quantified in vitro using the fluorescent probe 2′, 7′-dichlorofluorescein diacetate (DCFH-DA, KeyGEN, CHN), and the fluorescent intensity was then measured with a fluorescent microscope (Carl Zeiss) and flow cytometry. Data were analyzed using ImageJ and FlowJo Software. To measure the ROS level in the retina, 3-nitrotyrosine proteins were detected in retinal lysates using the Oxiselect Nitrotyrosine Kit (Cell Biolabs, Inc., USA) according to the manufacturer’s instructions. Ten thousand cells were collected and analyzed in each flow cytometric assay. Six independent assays were performed.

### Quantification of pro-inflammatory cytokines

The production of IL-1β and IL-18 was measured in the culture supernatant of primary microglia and retina lysates using commercially available ELISA kits (eBioscience, Vienna, Austria). Flow cytometric analysis was also performed using a IL-1β monoclonal antibody (FITC, eBioscience) and an IL-18 monoclonal antibody (PE, eBioscience) following standard protocols. Data were analyzed by FlowJo Software. Ten thousand cells were collected and analyzed in each flow cytometric assay. Six independent assays were performed.

### Statistical analysis

Data were presented as the mean ± standard deviation (SD). One-way analysis of variance (ANOVA) was performed, followed by Bonferroni’s post hoc test using SPSS Software, version 17 (SPSS Inc., USA). All statistical tests were two-tailed, and a *P*-value less than 0.05 were considered statistically significant.

## Supplementary information

Supplementary information
