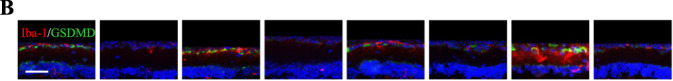# Correction to: LncRNA H19 initiates microglial pyroptosis and neuronal death in retinal ischemia/reperfusion injury

**DOI:** 10.1038/s41418-022-01105-w

**Published:** 2022-12-13

**Authors:** Peixing Wan, Wenru Su, Yingying Zhang, Zhidong Li, Caibin Deng, Jinmiao Li, Nan Jiang, Siyu Huang, Erping Long, Yehong Zhuo

**Affiliations:** 1 State Key Laboratory of Ophthalmology, Zhongshan Ophthalmic Center, Sun Yat-sen University, Guangzhou, 510060 China; 2 Department of Molecular, Cellular, and Developmental Biology, University of Michigan, Ann arbor, MI 48109 USA

Correction to: *Cell Death & Differentiation* 10.1038/s41418-019-0351-4, published online 24 May 2019

The original version of this article unfortunately contained an error in figure 5B. The corrected figure can be found below. This error does not affect the results, discussion, or conclusions. The authors sincerely apologize to the editors and readers for any inconvenience or confusion.