# Supplementary material for: LncRNA H19 initiates microglial pyroptosis and neuronal death in retinal ischemia/reperfusion injury
Source: Cell Death Differ. 2019 May 24;27(1):176–91. doi: 10.1038/s41418-019-0351-4 (PMC7206022; doi:10.1038/s41418-019-0351-4)
Supplement: Supplementary file 1 — Supplementary information [file 41418_2019_351_MOESM1_ESM.pdf]

<u>Supplementary information</u>

Table of Contents

1. Supplementary Figures……………………………………………………………..…..…2

2. Supplementary Tables…………………………………………………………….....…..27

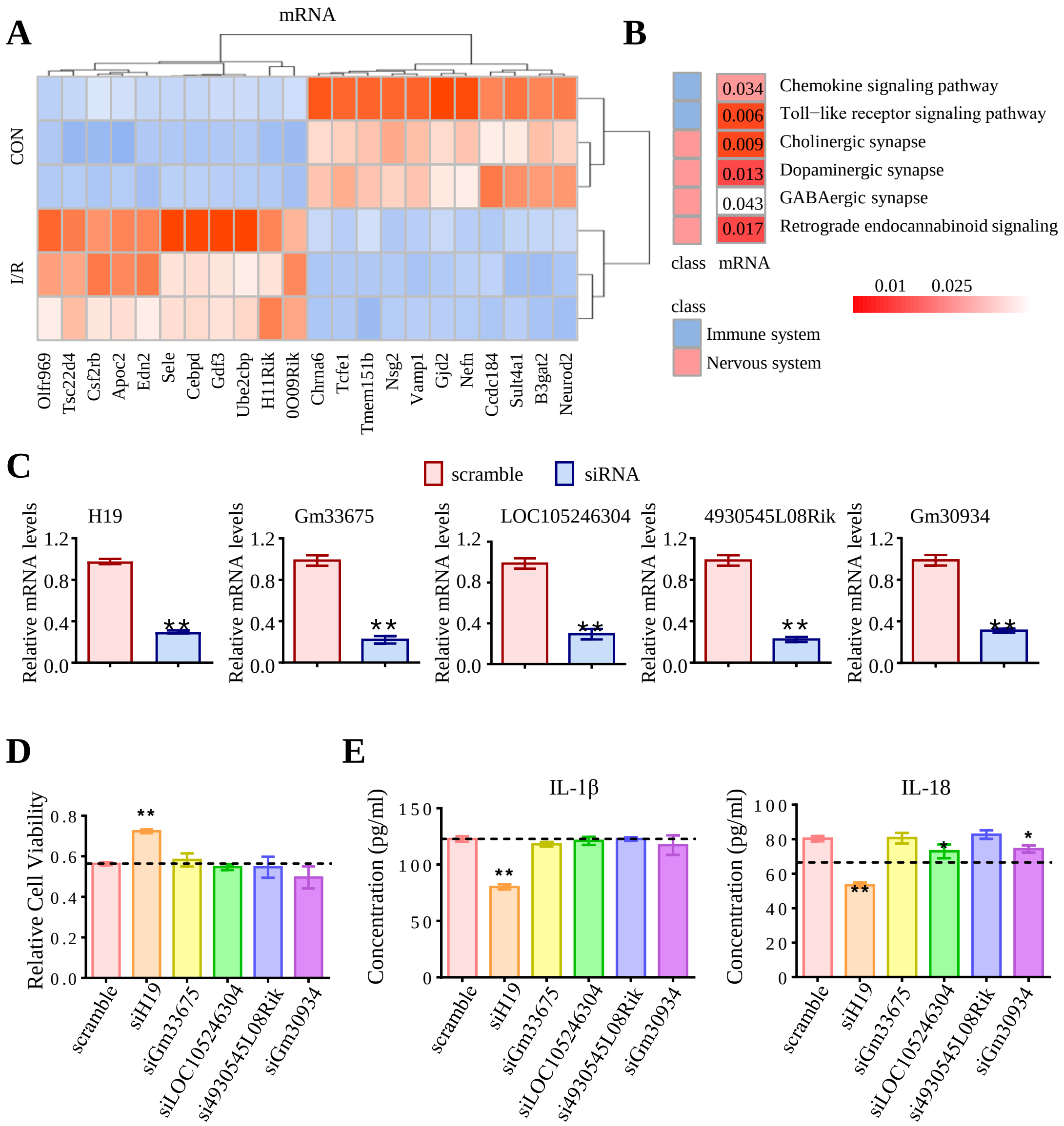

Figure S1. Identifying dysregulated lncRNAs in I/R

A. The most aberrantly expressed mRNAs in I/R injured retinas were listed in the heat map.

B. Panther analysis showed that dysregulated mRNAs mainly participated in neuro-inflammation and neuronal damage during I/R injury.

C. As measured by real-time PCR, siRNAs effectively decreased the expression of their target lncRNAs to an average of 31% compared with scramble.

D. Only H19 knockdown significantly protected the viability of RGCs against OGD/R injury. SiRNAs of four other lncRNAs lacked efficacy in affecting the viability of RGCs in OGD/R injury.

E. As measured by ELISA, H19 knockdown effectively repressed IL-1β and IL-18 overproduction in supernatant of cultured microglia. SiRNA of LOC105246304 and Gm30834 prevented aberrant production of IL-18 in microglia but failed in IL-1β.

Data were represented as means ± SD (n=6). Compared with the scrambled siRNA: $^{*}P<0.05$, $^{**}P<0.01$. I/R, ischemia and reperfusion; OGD/R, oxygen-glucose deprivation and reperfusion; si, siRNA; RGC, retinal ganglion cell.

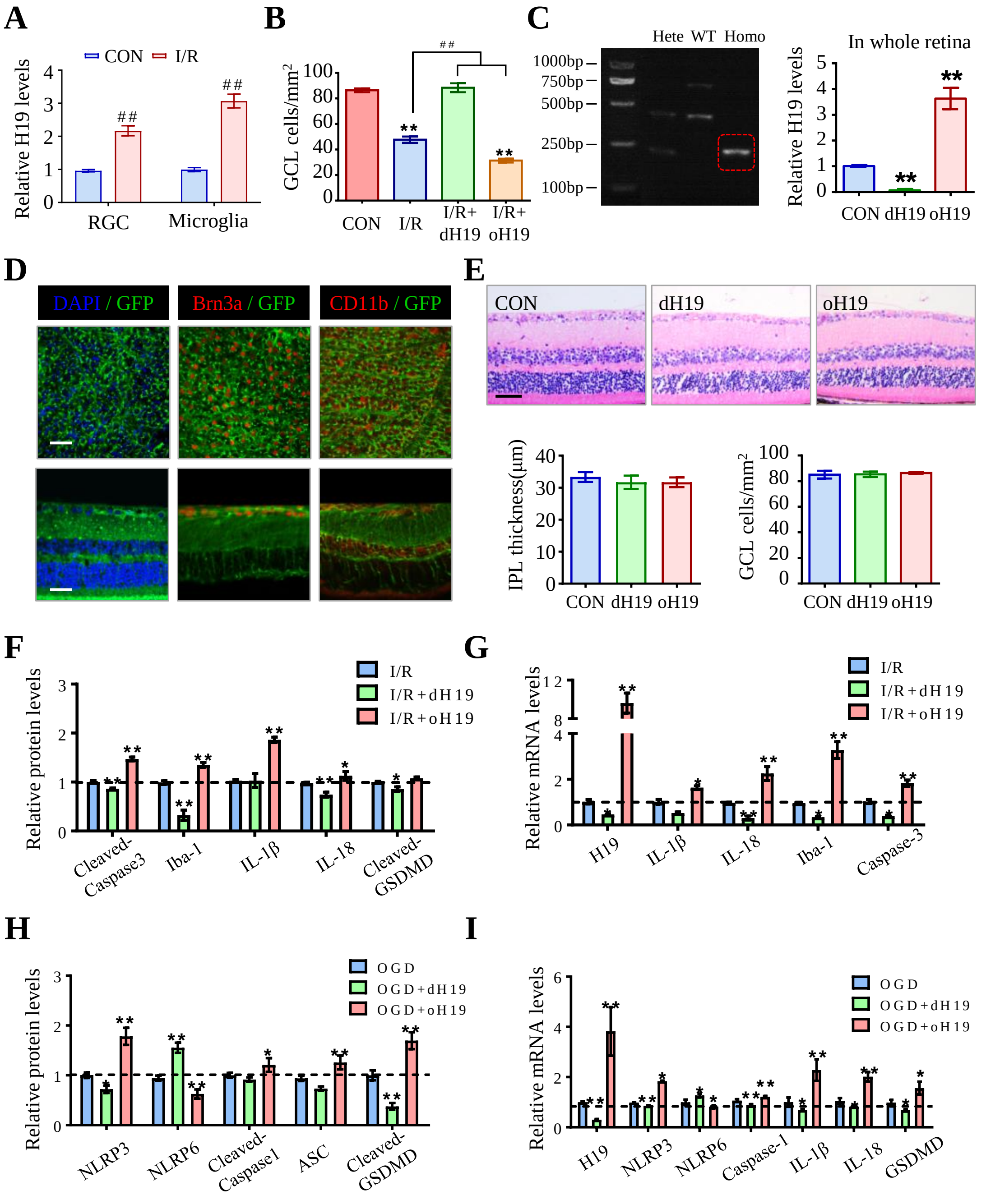

Figure S2. The anti-inflammatory role of H19 during I/R injury in the retina

A. RGCs and microglia were purified from I/R and control retinas to measure the H19 expression. As measured by real-time PCR, I/R significantly increased H19 levels in both RGCs and microglia. Compared with the normal control (CON): $^{\#\#}P$<0.01.

B. Cell density in the GCL significantly decreased in response to I/R, which was further reduced by H19 overexpression. H19-null retinas effectively resisted I/R-induced cell loss

in the GCL. Compared with the normal control (CON): $^{**}P<0.01$. Compared with the I/R retina: $^{\#\#}P<0.01$.

C. As measured by qPCR, mice homozygous for H19 knockout exhibited a single band in 240 bp (left). Consistently, H19 expression was almost eliminated in dH19 retinas (10.0 ± 1.1% vs CON) and greatly increased in oH19 retinas (363.3 ± 40.4% vs CON) as measured by real-time PCR. Compared with the normal control (CON): $^{**}P<0.01$.

D. Six weeks after GFP-lentivirus injection, retinas were flat-mounted or sliced to determine the transfection efficacy. GFP was expressed in the whole retina and especially accumulated in the GCL, IPL, and INL, where RGC and microglia localized. The transfection efficacy in RGCs and microglia was confirmed by co-labeling with their specific markers, Brn3a and CD11b, respectively.

E. Histological structures of dH19 and oH19 retinas exhibited no significant difference compared with wild type, especially in IPL thickness and cell density of the GCL.

F. As presented in immunoblot assay, H19 depletion effectively prevented I/R-induced caspase-3 cleavage, which was aggravated by oH19. The retina of H19-null mice also exhibited lower protein levels of Iba-1, cleaved-GSDMD, IL-1β, and IL-18. Compared with the I/R retinas: $^{*}P<0.05$, $^{**}P<0.01$.

G. As measured by real-time PCR, H19 knockout inhibited expression of pro-inflammatory cytokines, IL-1 β and IL-18. The I/R induced up-regulation of Iba-1 and caspase-3 was prevented by H19 knockout and augmented by H19 overexpression. Compared with the I/R retinas: $^{*}P<0.05$, $^{**}P<0.01$.

H. In primary microglia, H19 overexpression up-regulated the protein levels of NLRP3, cleaved-caspase-1, and cleaved- GSDMD, with decreased NLRP6 expression. However, H19 deficient retinas expressed less NLRP3 and active forms of caspase-1 and GSDMD. Compared with the OGD/R group (OGD): $^{*}P<0.05$, $^{**}P<0.01$.

I. As measured by real-time PCR, H19 knockout prevented the over-production of pro-inflammatory cytokines in microglia by rebalancing NLRP3/6 expression. H19 overexpression increased the mRNA levels of NLRP3, caspase-1, and GSDMD, with

decreased NLRP6 expression. Compared with the OGD/R group (OGD): *P<0.05, **P<0.01.

Scale bar = 100 μm. Data were represented as means ± SD (n=6). Hete, heterogynous; WT, wild type; Homo, homozygous; GCL, ganglion cell layer; IPL, inner plexiform layer; INL, inner nucleus layer; I/R, ischemic and reperfusion; dH19, H19 knockout; oH19, H19 overexpression; OGD/R, oxygen-glucose deprivation and reperfusion.

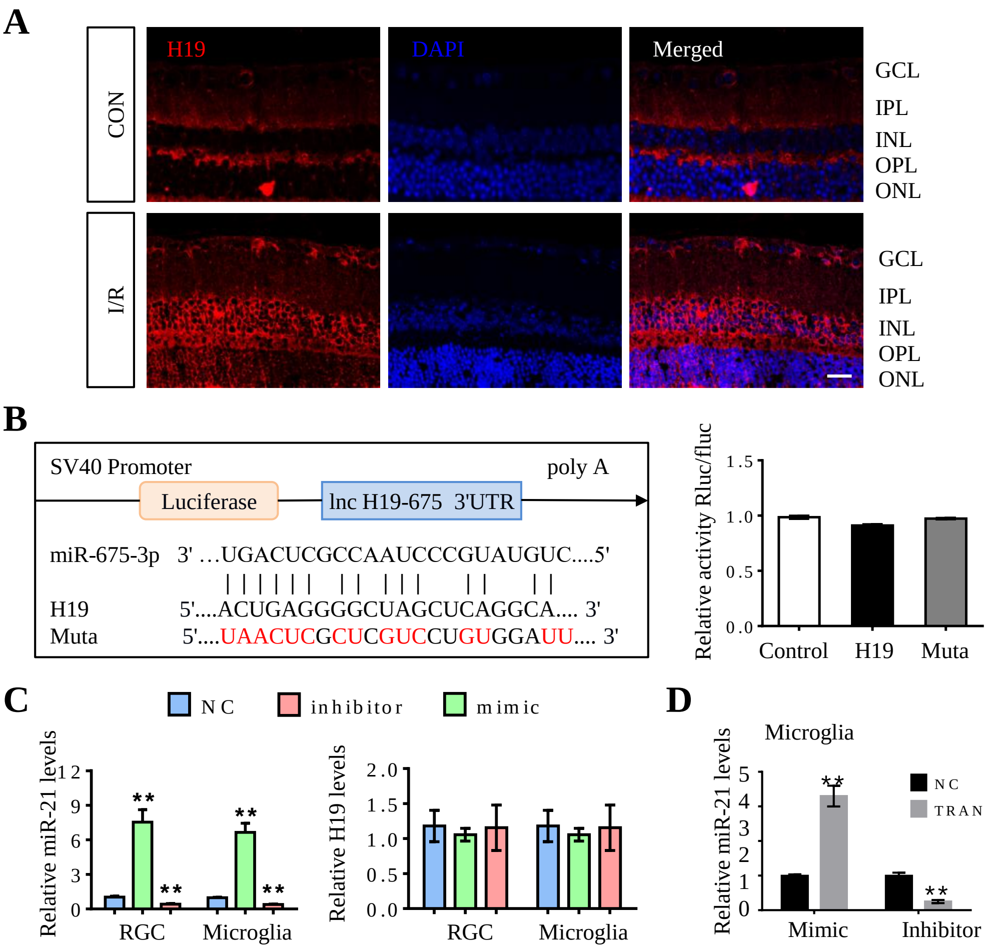

Figure S3. H19 functioned by sponging miR-21

A. FISH analysis was recruited to test the subcellular localization of H19 in retina. As presented as red signal, H19 was mainly distributed in the cytoplasm instead of nucleus among retinal cells. I/R injury increased H19 expression in both RGCs and microglia compared with normal control.

B. Compared to control non-coding RNA (Control), miR-675 failed to reduce luciferase activity for not binding to the H19 transcript. Mutations in the binding site of the H19 transcript (red marks) had little effect on luciferase activity. Data were represented as means ± SD (n=3).

C. Comparable copy numbers of H19 and miR-21 were detected, demonstrating a negative correlation between miR-21 and H19 in both primary RGCs and microglia. Data were represented as means ± SD (n=6). Compared with the normal control (NC): ***P*<0.01.

D. In primary microglia, the miR-21 mimic effectively elevated the level of miR-21. In miR-21 inhibitor transfected microglia, miR-21 level was hugely repressed as measured by real-time PCR. Data were represented as means ± SD (n=6). Compared with the normal control (NC): **P<0.01.

Scale bar = 100 μm. FISH, fluorescence in situ hybridization; TRAN, transfection.

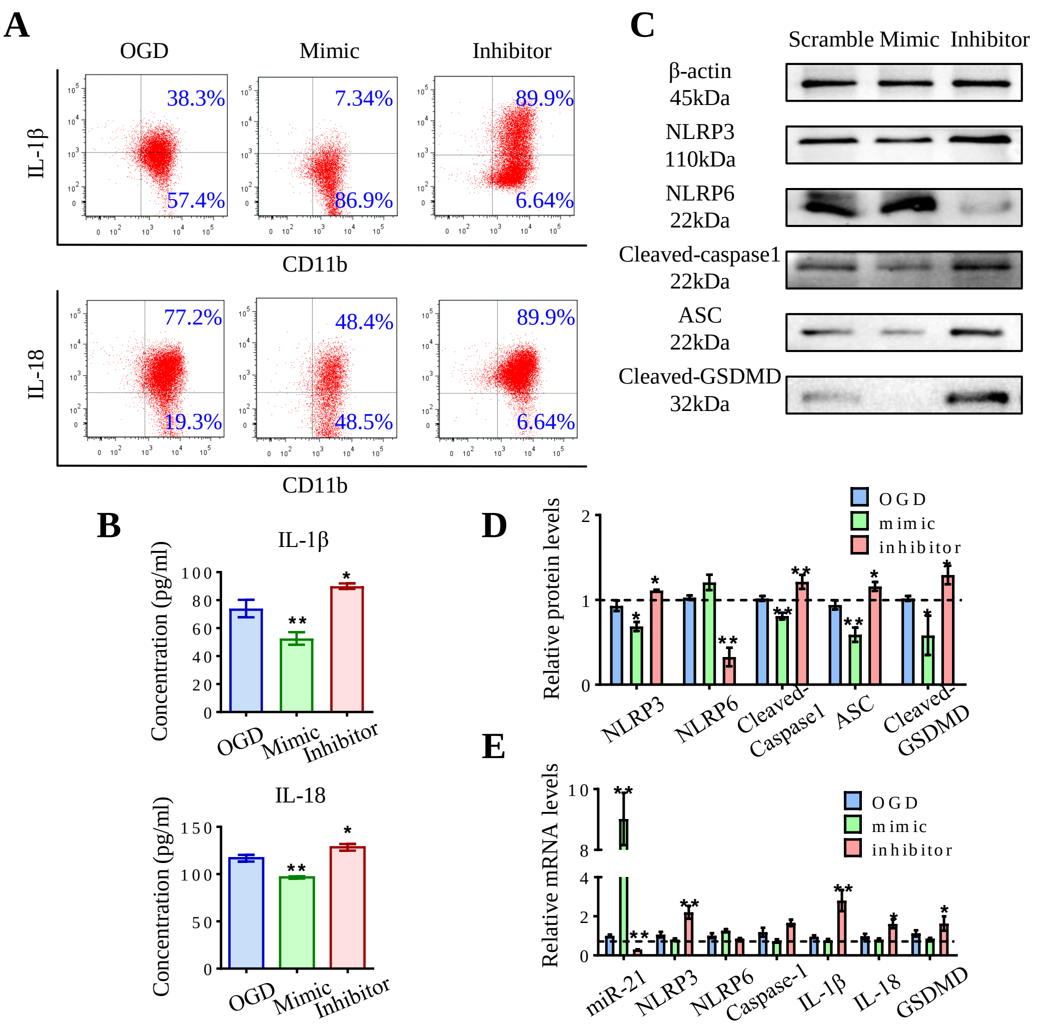

Figure S4. H19 sponged miR-21 to regulate neuro-inflammation in primary microglia

A and **B.** As measured by flow cytometry and ELISA, OGD/R significantly increased the production of IL-1β and IL-18 in cultured microglia. MiR-21 mimic inhibited the overproduction of these pro-inflammatory cytokines. The miR-21 inhibitor further aggregated OGD/R-induced neuro-inflammation.

C and **D.** OGD/R damage significantly suppressed NLRP6 protein production in microglia, and activated NLRP3 inflammasome. The imbalanced activation of NLRP3/6 inflammasome was aggravated by miR-21 knockdown and attenuated by the miR-21 mimic. The relative level of each target protein was normalized to β-actin from the same sample.

E. In primary microglia, the expression of NLRP3 was inhibited by miR-21 mimic, which induced NLRP6 transcription. MiR-21 mimic also prevented mRNA levels of caspase-1, IL-1β, IL-18, and GSDMD.

Data were represented as means ± SD (n=6). Compared with the OGD/R group (OGD): *P<0.05, **P<0.01. OGD/R, oxygen-glucose deprivation and reperfusion.

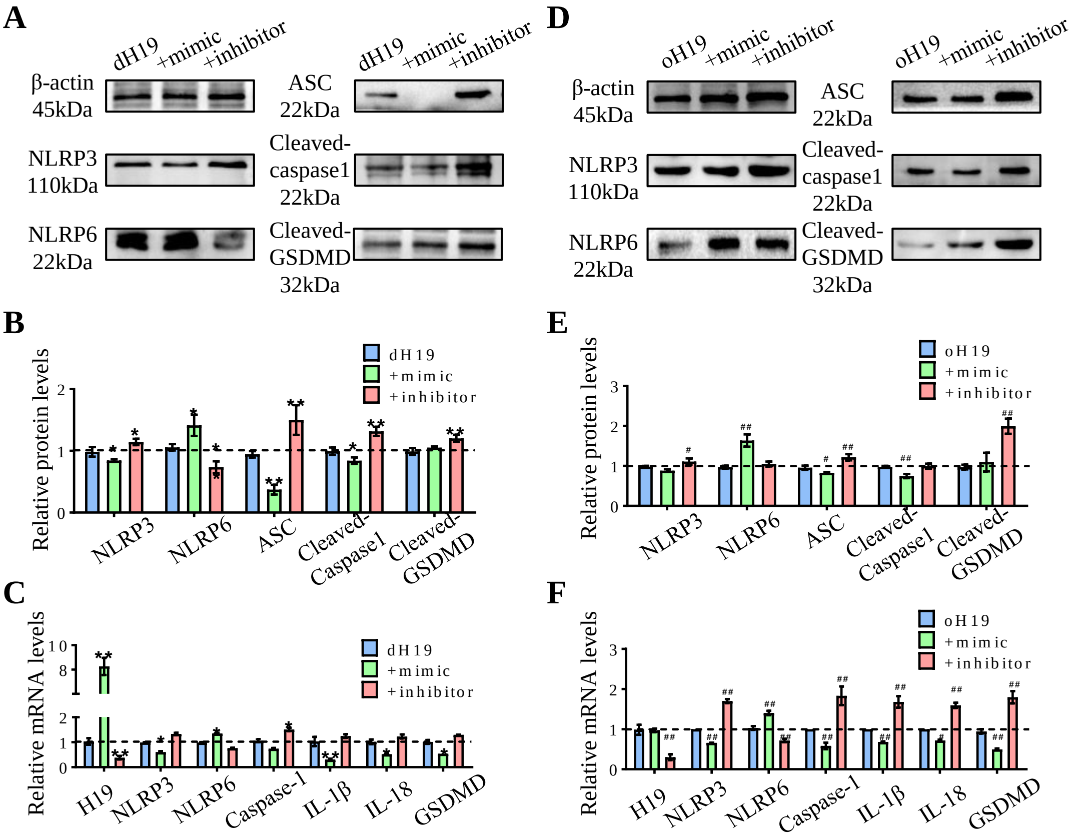

Figure S5. H19 regulate neuro-inflammation via NLRP3/NLRP6 inflammasomes balance

A-C. The miR-21 mimic further enhanced the anti-inflammatory effect of H19 depletion by resetting the balance between NLRP3 and NLRP6 inflammasomes, and inhibiting caspase-1 activation and GSDMD cleavage. On the contrary, the miR-21 inhibitor activated NLRP3, caspase-1, and GSDMD cleavage with decreased NLRP6 inflammasome as measured by Western blot and PCR.

D-F. The miR-21 inhibitor further augmented the pro-inflammatory effect of oH19 by reciprocally activating NLRP3 and NLRP6 inflammasomes as measured by Western blot and PCR. However, the miR-21 mimic partially eliminated oH19-induced damage by inhibiting caspase-1 activation, resetting the NLRP3/6 inflammasomes balance, and decreasing GSDMD.

Data were represented as means ± SD (n=6). The relative level of each target protein was normalized to β-actin from the same sample (taken as 1.0). Compared with the dH19 group: **P*<0.05, ***P*<0.01. Compared with oH19 microglia: $^{\#}P<0.05$, $^{\#\#}P<0.01$. dH19, H19

knockout; oH19, H19 overexpression; ASC, apoptosis-associated speck-like protein containing a CARD.

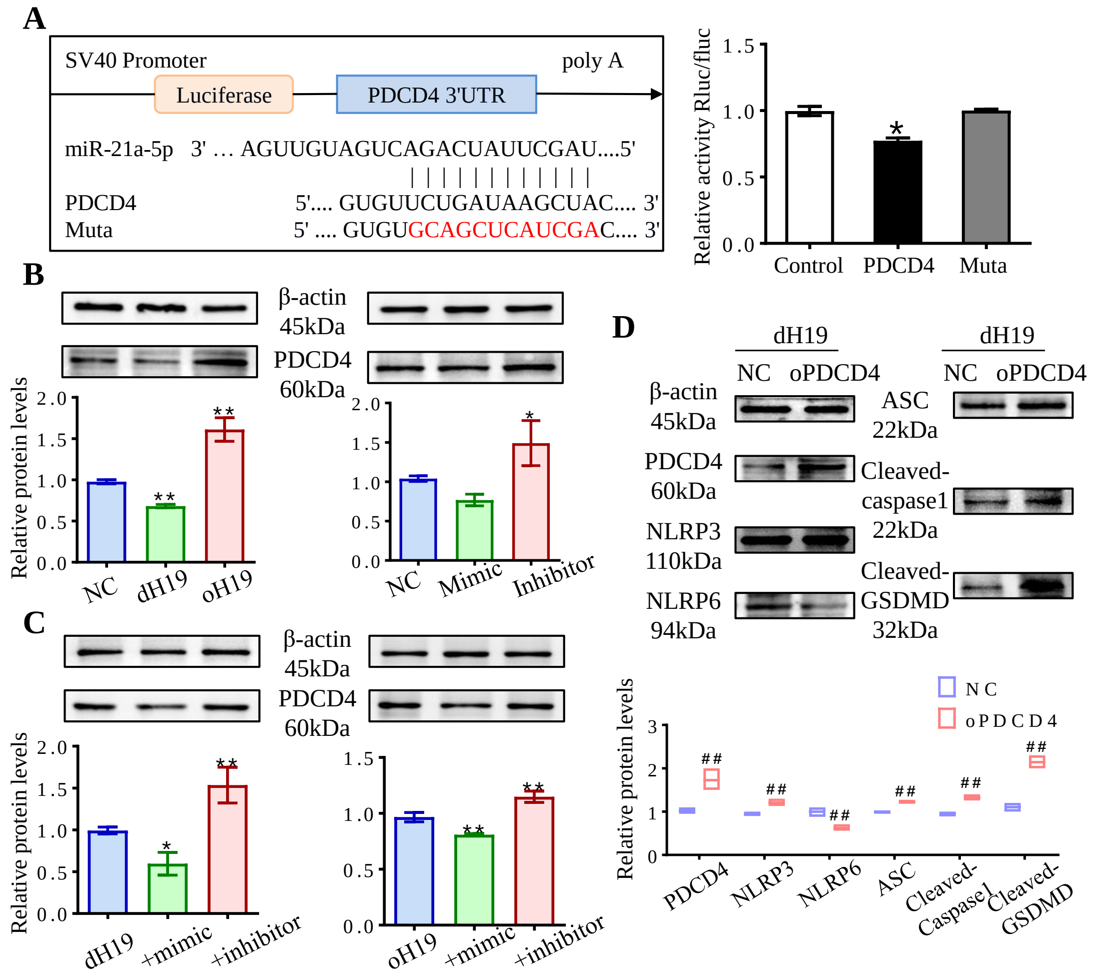

Figure S6. H19 competed with PDCD4 for miR-21 in ceRNET

A. Luciferase reporter assay was recruited to test the direct binding between miR-21 and PDCD4.The combination between miR-21 mimic and PDCD4 greatly decreased the luciferase activity. Mutations in their binding site (red) inhibited the decrease of luciferase activity, indicating that PDCD4 is the target gene of miR-21. Compared with control RNA: **P*<0.05.

B. PDCD4 decreased in response to either H19 knockout or miR-21 up-regulation as measured in protein levels. H19 overexpression and the miR-21 inhibitor significantly increased PDCD4 expression. Compared with normal control: **P*<0.05, ***P*<0.01.

C. MiR-21 mimic further decreased protein level of PDCD4, which was increased by oH19 together with miR-21 knockdown. Compared with dH19 or oH19: **P*<0.05, ***P*<0.01.

D. PDCD4 overexpression almost completely blocked the anti-inflammatory effects of dH19 as measured by Western blot. These all attributed to PDCD4 activated NLRP3

inflammasome, caspase-1, and GSDMD and suppressed NLRP6 in microglia. Conpared with normal control: $^{\#\#}P<0.01$.

Data were represented as means ± SD (n=6). The relative level of each target protein was normalized to β-actin from the same sample (taken as 1.0). NC, normal control; dH19, H19 knockout; oH19, H19 overexpression; oPDCD4, PDCD4 overexpression; ASC, apoptosis-associated speck-like protein containing a CARD.

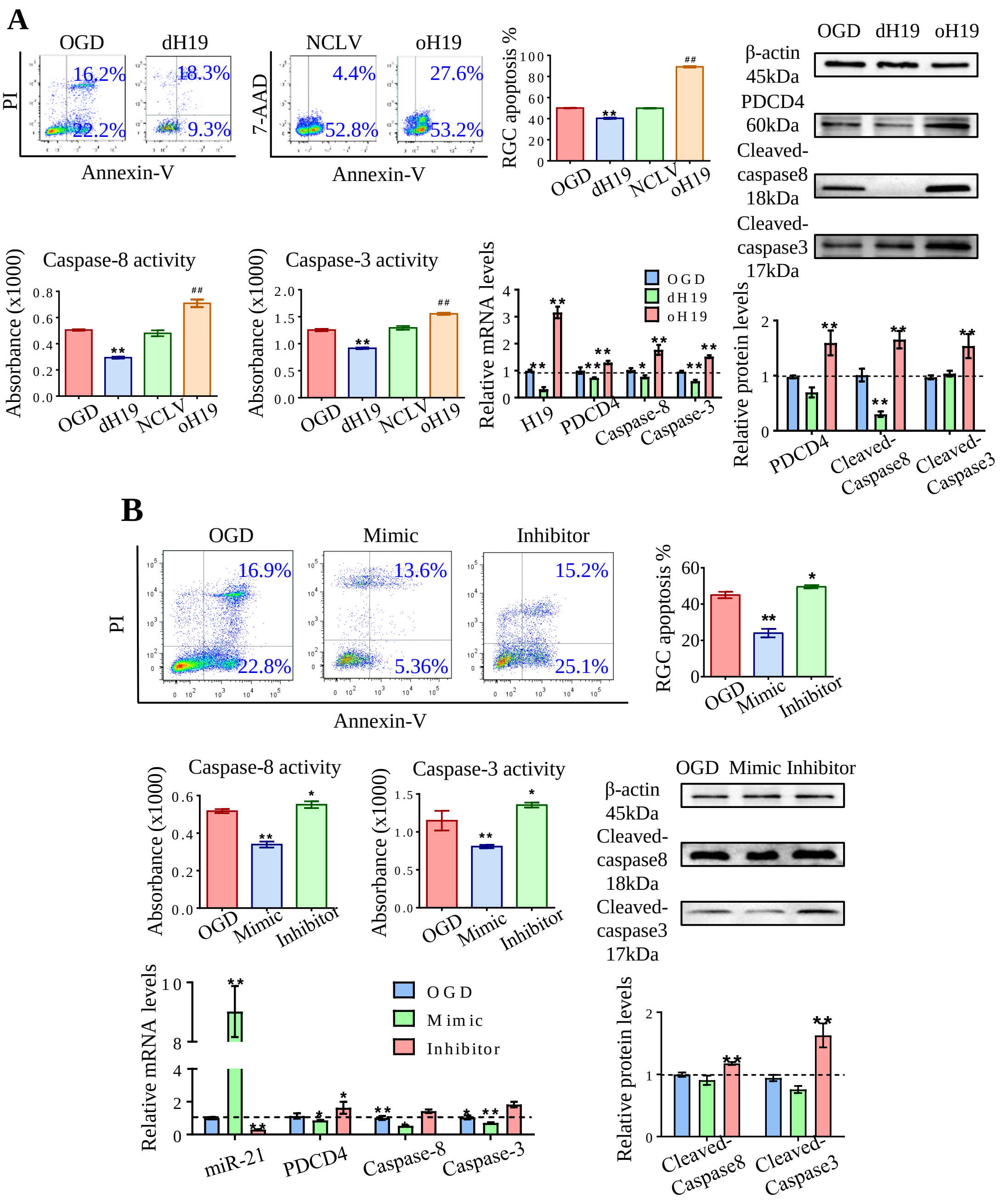

Figure S7. H19 and miR-21 participated in neuroprotection

A. The number of apoptotic RGCs (marked by Annexin V+) significantly decreased by H19 knockout. By contrast, H19 overexpression dramatically decreased RGC survival, which was caused by activation of caspase-8 and caspase-3.

B. The miR-21 mimic inhibited RGC apoptosis by inhibiting activation of caspase-mediated apoptotic signaling. The miR-21 inhibitor increased the RGC apoptotic rate.

Data were represented as means ± SD (n=6). The relative level of each target protein was normalized to β-actin from the same sample (taken as 1.0). Compared with the OGD group: **P*<0.05, ***P*<0.01. Compared with NCLV transfected cells: ##*P*<0.01. OGD/R, oxygen-glucose deprivation and reperfusion; dH19, H19 knockout; oH19, H19 overexpression; NCLV, normal control of lentivirus.

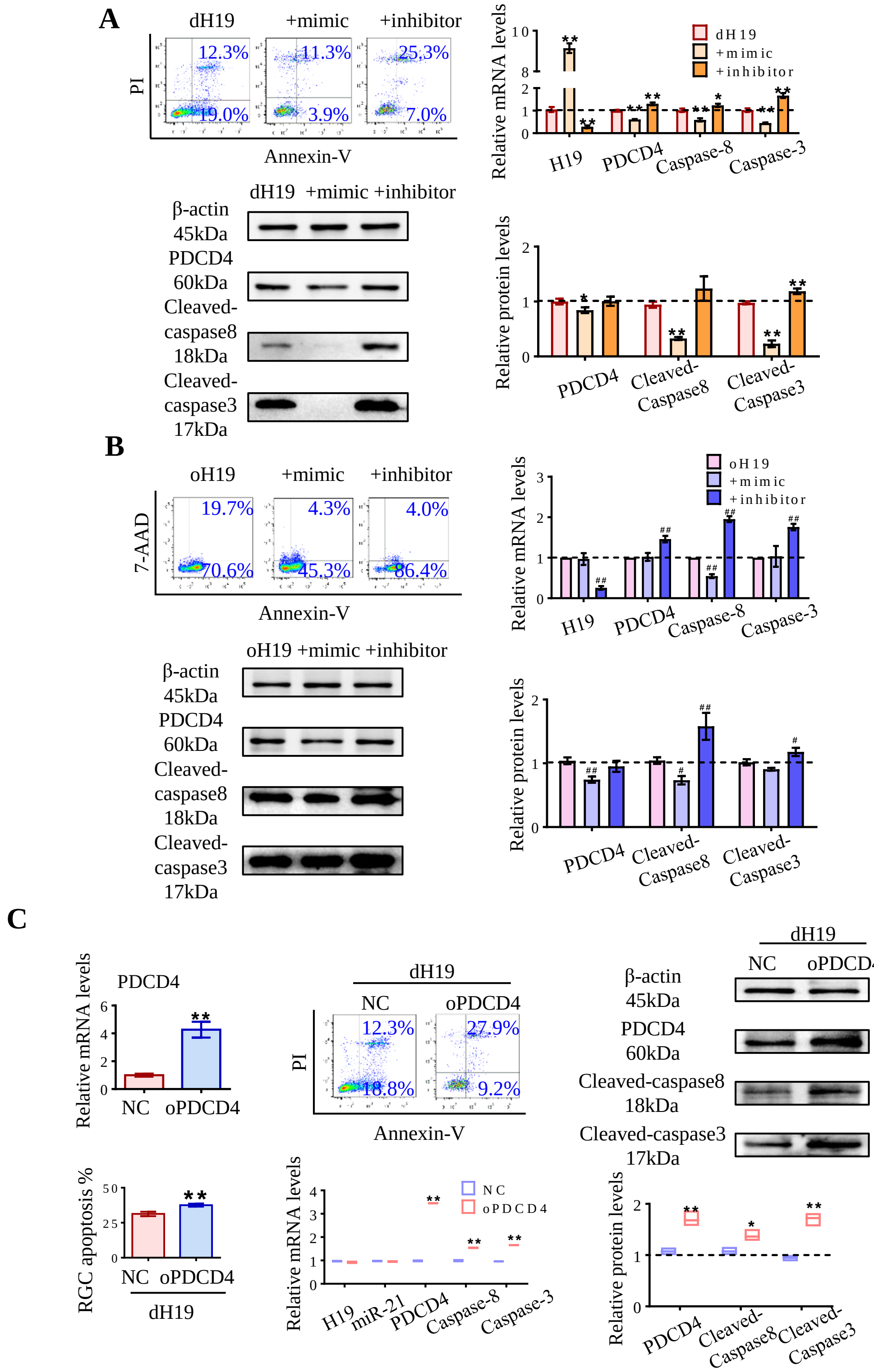

Figure S8. H19/miR-21/PDCD4 ceRNET directly regulated RGC apoptosis via caspase signaling

A. H19 knockout exerted anti-apoptotic effects in primary RGCs, which were further protected by the miR-21 mimic and abolished by the inhibitor.

B. H19 overexpression promoted RGCs apoptosis by activating PDCD4 and caspase-mediated apoptotic signal in response to OGD/R. MiR-21 down-regulation further aggravated caspase activation and apoptosis in oH19 RGCs.

C. The neuroprotective effect of dH19 was greatly reversed by PDCD4 overexpression by abberantly activated caspase-8 and caspase-3.

Data were represented as means ± SD (n=6). The relative level of each target protein was normalized to β-actin from the same sample (taken as 1.0). Compared with dH19 cells: *P<0.05, **P<0.01. Compared with oH19 cells: #P<0.05, ##P<0.01. dH19, H19 knockout; oH19, H19 overexpression; NC, normal control of lentivirus; oPDCD4, PDCD4 overexpression.

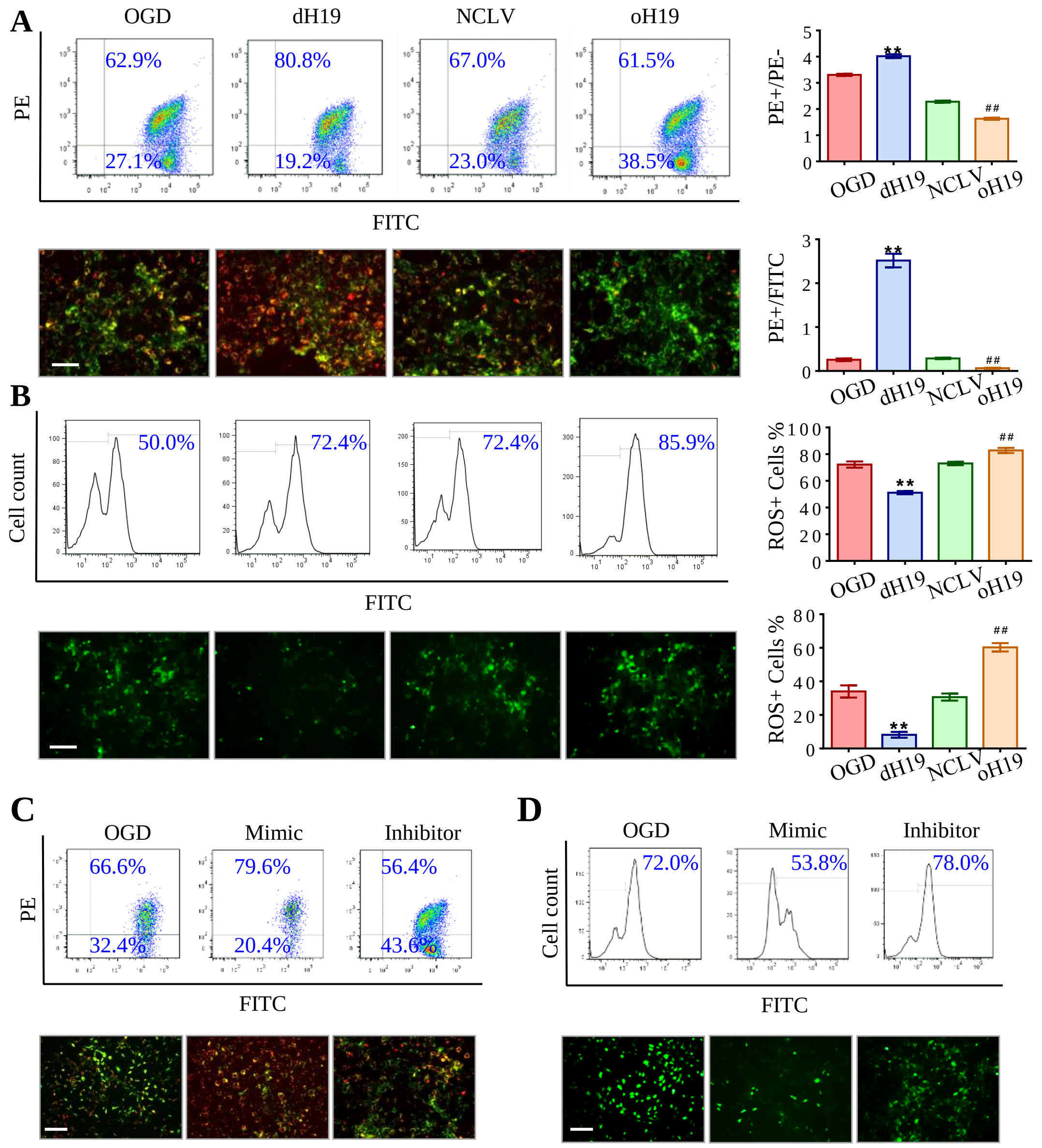

Figure S9. H19 and miR-21 regulated mitochondrial function and ROS accumulation in I/R damage

A. OGD/R decreased MMP in primary microglia, which was marked by a shift in fluorescence from red to green. MMP collapse was rescued by dH19 and augmented by oH19 as measured by flow cytometry and fluorescent assay.

B. ROS over-loaded microglia (FITC+) were reduced by H19 knockout and increased by H19 overexpression as measured by both flow cytometry and fluorescent assay.

C. In primary microglia, OGD/R-induced MMP collapse was reserved by the miR-21 mimic, and the miR-21 inhibitor augmented the decrease in the PE/FITC ratio.

D. MiR-21 up-regulation effectively decreased intracellular ROS accumulation, which was increased by the miR-21 inhibitor.

Scale bar = 100 μm. Data were represented as means ± SD (n=6). Compared with the OGD group: ***P*<0.01. Compared with NCLV transfected microglia: ##*P*<0.01. MMP, mitochondrial membrane potential; OGD/R, oxygen-glucose deprivation and reperfusion; dH19, H19 knockout; oH19, H19 overexpression; NCLV, normal control of lentivirus.

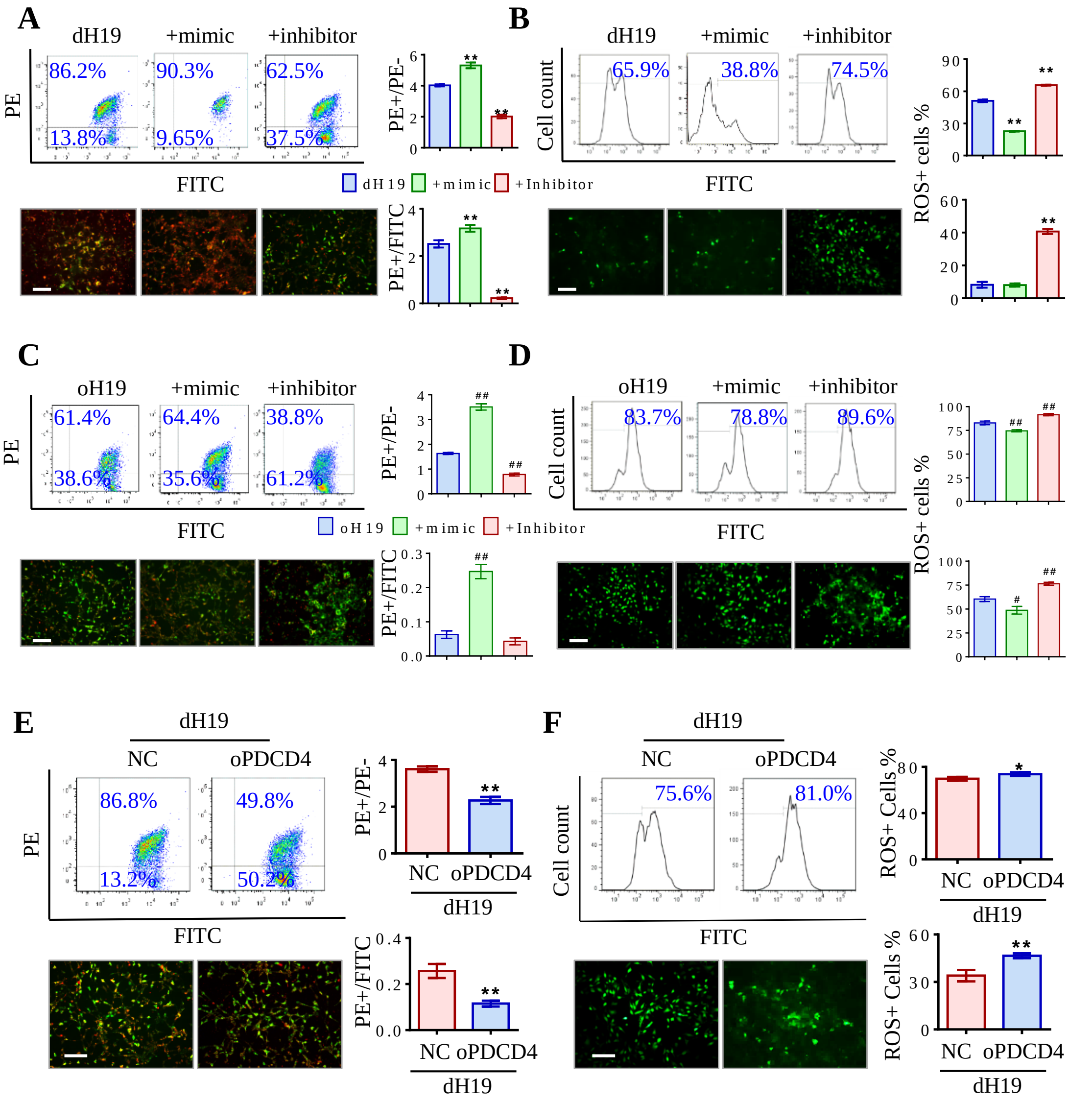

Figure S10. H19/miR-21/PDCD4 ceRNET induced mitochondrial dsyfunction in I/R damage

A. The miR-21 mimic further protected mitochondrial function by increasing PE+/PE- in H19 deficient microglia, which was damaged by the miR-21 inhibitor.

B. H19 knockout inhibited intracellular ROS accumulation (FITC+) in response to OGD/R, which was further reduced by the miR-21 mimic and contradicted by the miR-21 inhibitor, as measured by flow cytometry and fluorescent assay.

C. In H19 over-expressed microglia, MMP collapse was ameliorated by the miR-21 mimic and augmented by its inhibitor.

D. ROS+ cells were further increased by the miR-21 inhibitor and reduced by the miR-21 mimic in the oH19 group.

E and **F.** PDCD4 overexpression mostly blocked the mitochondrial protective effect of dH19 against OGD/R. PDCD4 exerted mitochondrial damage by promoting MMP collapse and intracellular ROS accumulation.

Scale bar = 100 μm. Data were represented as means ± SD (n=6). Compared with the dH19 group: **P*<0.05, ***P*<0.01. Compared with the oH19 group: #*P*<0.05, ##*P*<0.01. NC, normal control of lentivirus; dH19, H19 knockout; oH19, H19 overexpression; oPDCD4, PDCD4 overexpression.

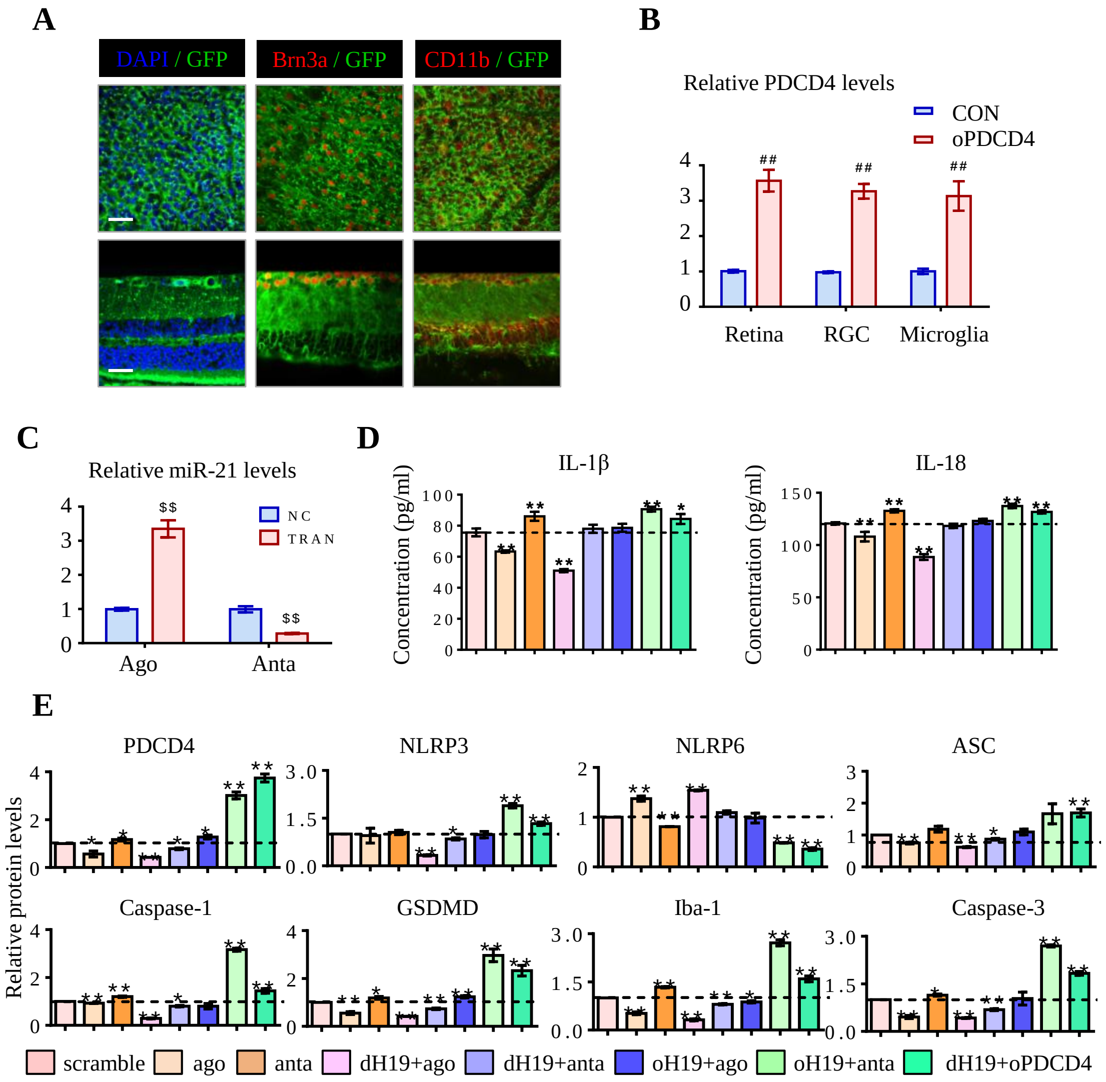

Figure S11. H19/miR-21/PDCD4 worked within the ceRNET to regulate I/R-induced retinal damage

A. PDCD4 gene was loaded in a GFP-lentivirus for overexpression. GFP was expressed in the flat-mounted and sliced retinas. The overexpression efficacy in both RGCs and microglia was confirmed by co-labeling with their specific markers Brn3a and CD11b, respectively.

B. As measured by real-time PCR, PDCD4 expression was increased by 3.8 fold in the entire retina and purified cells. Compared with control lentivirus transfected retinas (CON): ##*P*<0.01.

C. Agomir effectively elevated the miR-21 level by 4 fold in the retina of mice. And miR-21 antagomir significantly decreased miR-21 to 28.2% compared with the scrambled siRNA. Compared with the scrambled siRNA (NC): $^{\$\$}P<0.01$.

D. As measured in supernatant of microglia, H19 knockout and miR-21 mimic worked in cooperation to inhibit the production of IL-1β and IL-18. H19 overexpression and miR-21 down-regulation augmented I/R-induced overproduction of these two inflammatory cytokines.

E. As measured by real-time PCR, H19 excision significantly prevented the imbalanced activation of NLRP3/NLRP6 inflammasomes and pyroptosis markers, including caspase-1 and GSDMD. H19 knockout exerted an anti-inflammatory effect that was further enhanced by miR-21 mimic. H19 overexpression triggered increase of the NLRP3 gene and pyroptosis-related genes, with reciprocal suppression of NLRP6. H19 overexpression induced gene dysregulation was ameliorated by miR-21 up-regulation and augmented by its down-regulation.

Scale bar = 100 μm. Data were represented as means ± SD (n=6). NC, scrambled siRNA; TRAN, transfection; CON, retinas transfected with control lentivirus; dH19, H19 knockout; oH19, H19 overexpression; oPDCD4, PDCD4 overexpression; ago, agomir of miR-21; anta, antagomir of miR-21.

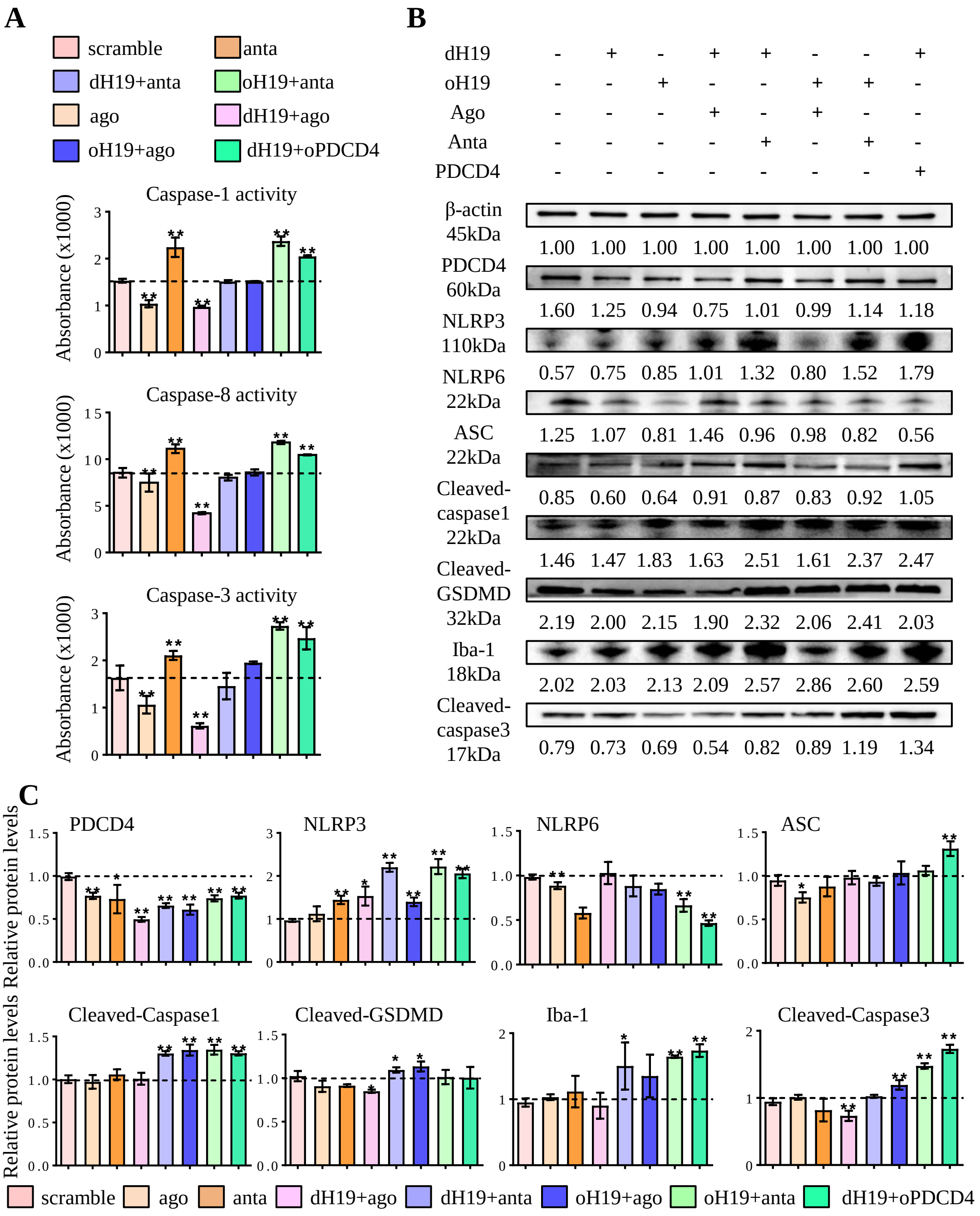

Figure S12. Specific role of H19/miR-21/PDCD4 ceRNET in I/R induced retinal damage

A. H19 knockout inhibited the activity of pro-inflammatory and apoptotic caspases, including caspase-1, caspase-8 and caspase-3. But this protective effect was abolished by PDCD4 overexpression in retinas. Meanwhile, H19 overexpression activated the caspase cascade, which was further irritated by miR-21 antagomir and reversed by miR-21 agomir.

B and **C.** Imbalanced activation of NLRP3/NLRP6 inflammasomes, caspase signals, and GSDMD cleavage was silenced in H19 knockout retinas and further decreased with miR-21. H19 depletion induced protective effect was abolished by miR-21 antagomir and PDCD4 overexpression *in vivo*. By contrast, H19 overexpression triggered expression of the NLRP3 inflammasome and activation of caspase-1 and GSDMD with decrease of NLRP6. These detrimental effects were ameliorated by miR-21 up-regulation and augmented by miR-21 down-regulation. The relative level of each target protein was normalized to β-actin from the same sample (taken as 1.0).

Data were represented as means ± SD (n=6). Compared with the I/R+scramble group: *$P<0.05$, **$P<0.01$. dH19, H19 knockout; oH19, H19 overexpression; oPDCD4, PDCD4 overexpression; ago, agomir of miR-21; anta, antagomir of miR-21.

Table S1. Primary antibodies and dilutions.

Primary antibody	Manufacture	Product size	Dilution
β-actin antibody	CST	45 kDa	WB(1:1000)
PDCD4 antibody	CST	60 kDa	WB(1:1000)
ASC antibody	CST	22 kDa	WB(1:1000)
Cleaved caspase-1 antibody	CST	22 kDa	WB(1:1000)
Cleaved caspase-3 antibody	CST	17, 19 kDa	WB(1:1000)
Cleaved caspase-8 antibody	CST	18, 43 kDa	WB(1:1000)
IL-1β	CST	17, 31 kDa	WB(1:1000)
IL-18	Abcam	22 kDa	WB(1:1000),
Iba-1 antibody	Abcam	17 kDa	WB(1:1000), IF (1:200)
GSDMD antibody	Sigma	32, 55kDa	WB(1:1000), IF (1:200)
NLRP3 antibody	NOVUS Biologicals	110 kDa	WB(1:1000)
NLRP6 antibody	Sigma	94 kDa	WB(1:1000)
IL-1β, PE	eBioscience	/	FC(1:500)
IL-18, FITC	eBioscience	/	FC(1:500)
CD11b antibody	BD Biosciences	170kDa	FC(1:500) IF(1:200)
Brn-3a antibody	SANTA CRUZ	43kDa	IF (1:500)

Table S2. Primer sequences used for real-time PCR

Primer	Sequence	Target size
β-actin_F	GCCAACCGTGAAAAGAT	173bp
β-actin_R	AGAGCATAGCCCTCGTAGAT	
ASC_F	AGACATGGGCTTACAGGA	257bp
ASC_R	CTCCCTCATCTTGTCTTGG	
NLRP3_F	GGTCCTCTTTACCATGTGCTTC	365bp
NLRP3_R	AAGTCATGTGGCTGAAGCTGTA	
NLRP6_F	AAACACCCTCACACCCAGAA	340bp
NLRP6_R	CCTGCCGGGTCATTTCTACA	
Caspase-1_F	TGAAAGAGGTGAAAGAATT	424bp
Caspase-1_R	TCTCCAAGACACATTATCT	
GSDMD_F	TGTTGTCAGGCATGGGAGAA	958bp
GSDMD_R	CTCTGTTCCAAGACGTGCTTCA	
Iba-1_F	TGGGAGTTAGCAAGGGAATG	120bp
Iba-1_R	AGACGCTGGTTGTCTTAGGC	
IL-1β_F	GGGCCTCAAAGGAAAGAATC	201bp
IL-1β_R	CTCTGCTTGTGAGGTGCTGA	
IL-18_F	CTGTACAACCGCAGTAATACGG	264bp
IL-18_R	ACTCCATCTTGTTGTGTCCTGG	
H19_F	GCAGGTAGAGCGAGTAGCTG	658bp
H19_R	TAGAGGCTTGGCTCCAGGAT	
PDCD4_F	AAAGACGACTGCGGAAAAATTCA	82bp
PDCD4_R	CTTCTAACCGCTTCACTTCCATT	
Caspase-3_F	TGTACGCGCACAAGCTAGAATT	97bp
Caspase-3_R	TGGAAAGTGGAGTCCAGGGAGA	
Caspase-8_F	CTCCGAAAAATGAAGGACAGA	193bp
Caspase-8_R	CGTGGGATAGGATACAGCAGA	

F, forward; R, reverse. Stem-loop primers of miR-21 were provided by RiboBio. Tech.
